# Conditional Diffusion–Augmented Cascaded Multi-View Attention BiLSTM for Non-Invasive Blood Glucose Estimation from Photoplethysmography

**DOI:** 10.3390/bioengineering13090974

**Published:** 2026-08-25

**Authors:** Chaofan Mo, Jianfeng He

**Affiliations:** Faculty of Information Engineering and Automation, Kunming University of Science and Technology, Kunming 650500, China; 20242104131@stu.kust.edu.cn

**Keywords:** non-invasive blood glucose estimation, photoplethysmography, conditional diffusion model, physiological signal augmentation, multi-view attention, bidirectional long short-term memory

## Abstract

Photoplethysmography (PPG)-based blood glucose estimation is attractive for non-invasive monitoring, but its development is constrained by limited paired PPG–glucose data and the weak representation of glucose-related waveform variations. This study proposes a framework combining conditional diffusion-based data augmentation with a cascaded multi-view attention bidirectional long short-term memory (BiLSTM) network. Blood glucose level, heart rate, and body mass index were used as physiological conditions to generate synthetic PPG segments, while channel and temporal attention together with cascaded BiLSTM layers were used to extract discriminative spatiotemporal features. A moth–flame optimization algorithm was employed to tune key hyperparameters. Experiments were conducted on a public dataset containing 67 PPG recordings from 23 participants. The framework was evaluated using subject-wise five-fold cross-validation, ensuring that all recordings from the same participant remained within a single fold. In the primary seed-42 analysis, the proposed method achieved a recording-level RMSE of 0.59 mmol/L, an MAE of 0.38 mmol/L, a MARD of 5.48%, and Clarke Zone A and A + B proportions of 97.0% and 100%, respectively; repeating the complete evaluation with seeds 2026 and 3407 produced closely similar recording-level results. These results suggest that physiologically conditioned generative augmentation may improve PPG-based blood glucose estimation in small-sample settings.

## 1. Introduction

Diabetes is a chronic metabolic disease affecting more than 500 million people worldwide, and its prevalence continues to rise. Given that there is currently no cure, effective disease management relies heavily on daily monitoring of blood glucose levels to guide insulin dosing, diet, and physical activity. Current mainstream traditional fingerstick methods can only provide intermittent readings; they are not only painful but also carry a slight risk of infection. These factors collectively reduce patient adherence to prescribed monitoring regimens. These limitations continue to drive research into non-invasive blood glucose monitoring technologies, with the goal of achieving pain-free, convenient, and effective continuous glucose monitoring [1,2,3,4,5].

Among the various non-invasive sensing methods explored to date (including near-infrared spectroscopy, bioimpedance, and sweat analysis), PPG stands out in particular. PPG is an optical technology that measures changes in blood volume within the microvascular bed by illuminating the skin and detecting changes in reflected or transmitted light [6]. This technology has been widely adopted in consumer-grade wearable devices for heart rate and blood oxygen saturation monitoring, indicating that its sensor hardware is inexpensive, compact, and well established. In the context of glucose estimation, however, PPG should not be interpreted as a direct glucose-specific optical sensor. The relationship between BGL and the PPG waveform is likely indirect and multifactorial, reflecting glucose-associated physiological and hemodynamic covariation together with effects of heart rate, blood pressure, tissue perfusion, skin temperature, and other optical or vascular confounders [7]. Accordingly, data-driven prediction from PPG represents the estimation of an association rather than direct optical measurement of glucose concentration.

An increasing number of studies have demonstrated the feasibility of estimating blood glucose levels using PPG. Zheng et al. developed a highly sensitive near-infrared PPG sensor based on a hybrid tin-lead perovskite photodetector. In their multi-participant evaluation involving 49 volunteers, the reported results were an RMSE of 2.38 mmol/L, a MARD of 6.93%, and 93% of predictions in Clarke Zone A [8]. Li et al. integrated the spatiotemporal features of ECG and PPG and employed weighted Choquet integration for multi-model fusion, achieving a prediction accuracy of 80.09% for Zone A and 99.49% for Zones A + B on the Parkes error grid in a 10-fold cross-validation (RMSE 1.49 mmol/L, MARD 13.42%) [9]. Şentürk et al. proposed a hybrid Mamba-MoE architecture, achieving 98.16% accuracy in Zone A and a low root mean square error (RMSE) on limited samples [10]. Other notable achievements include deep capsule networks, LSTM models based on blood volume pulse signals, and ensemble systems of machine learning regressors such as XGBoost [11,12,13,14,15,16]. Taken together, these studies support the feasibility of statistical BGL estimation from PPG-derived signals, but they do not establish that glucose produces a unique or directly measured optical signature in the PPG waveform.

More recently, Soliman et al. addressed the data-scarcity problem through cross-domain transfer learning: a parallel CNN–LSTM model was pretrained on a large PPG–blood-pressure dataset and then fine-tuned on the small Mazandaran PPG–BGL dataset, illustrating how hemodynamic representations learned from a data-rich physiological task can be transferred to glucose estimation [17]. In a related PPG modeling context, Msokar et al. developed an interpretable multi-domain feature-engineering framework for blood-pressure staging and emphasized rigorous preprocessing, leakage control, and transparent physiological features when working with small or imbalanced PPG datasets [18]. Although the latter study addresses blood pressure rather than glucose, it highlights methodological issues that are directly relevant to robust PPG-based physiological inference.

However, two major challenges continue to hinder the clinical application of PPG-based non-invasive blood glucose monitoring. The first is data scarcity. Obtaining high-quality, long-term records that pair PPG signals with synchronized reference blood glucose measurements is both costly and logistically extremely difficult. Consequently, most publicly available datasets include fewer than 100 participants, and individual recordings rarely cover the full range of blood glucose states encountered in daily life. Traditional data augmentation techniques—such as adding noise, time warping, or scaling—can only alter waveform morphology and cannot explicitly generate diverse waveform realizations conditioned on observed glucose levels and physiological variables while maintaining signal–label consistency [19]. The second challenge lies in modeling complexity. PPG signals are inherently nonlinear and non-stationary; the influence of glucose on the waveform is indirect and easily confounded by heart rate, blood pressure, skin temperature, and other hemodynamic variables. Shallow feature extraction methods and conventional neural network architectures often lack the ability to disentangle these superimposed effects and struggle to capture the long-range temporal dependencies inherent in dynamic blood glucose changes.

The main contributions of this study are as follows:

(1) A physiologically conditioned diffusion model is developed to generate diverse PPG waveform realizations according to blood glucose level, heart rate, and body mass index, thereby increasing waveform diversity around physiological states represented in the training data.
(2) Building on the CBHFF backbone, an improved cascaded multi-view attention BiLSTM network is developed. The original MLP-based channel attention is replaced with a lightweight local cross-channel Conv1D mechanism, while the dilated-convolution spatial branch is replaced with multi-head temporal self-attention. A coefficient-controlled multiplicative fusion strategy is further introduced to model joint channel–temporal importance. These architectural changes are evaluated jointly against the original CBHFF configuration; the present study does not assign the observed performance difference to any one internal modification in isolation.
(3) A complete training framework is constructed by integrating conditional data augmentation, multi-view temporal feature learning, and automatic hyperparameter optimization.

## 2. Methods

### 2.1. Overall Framework

**Figure 1:**
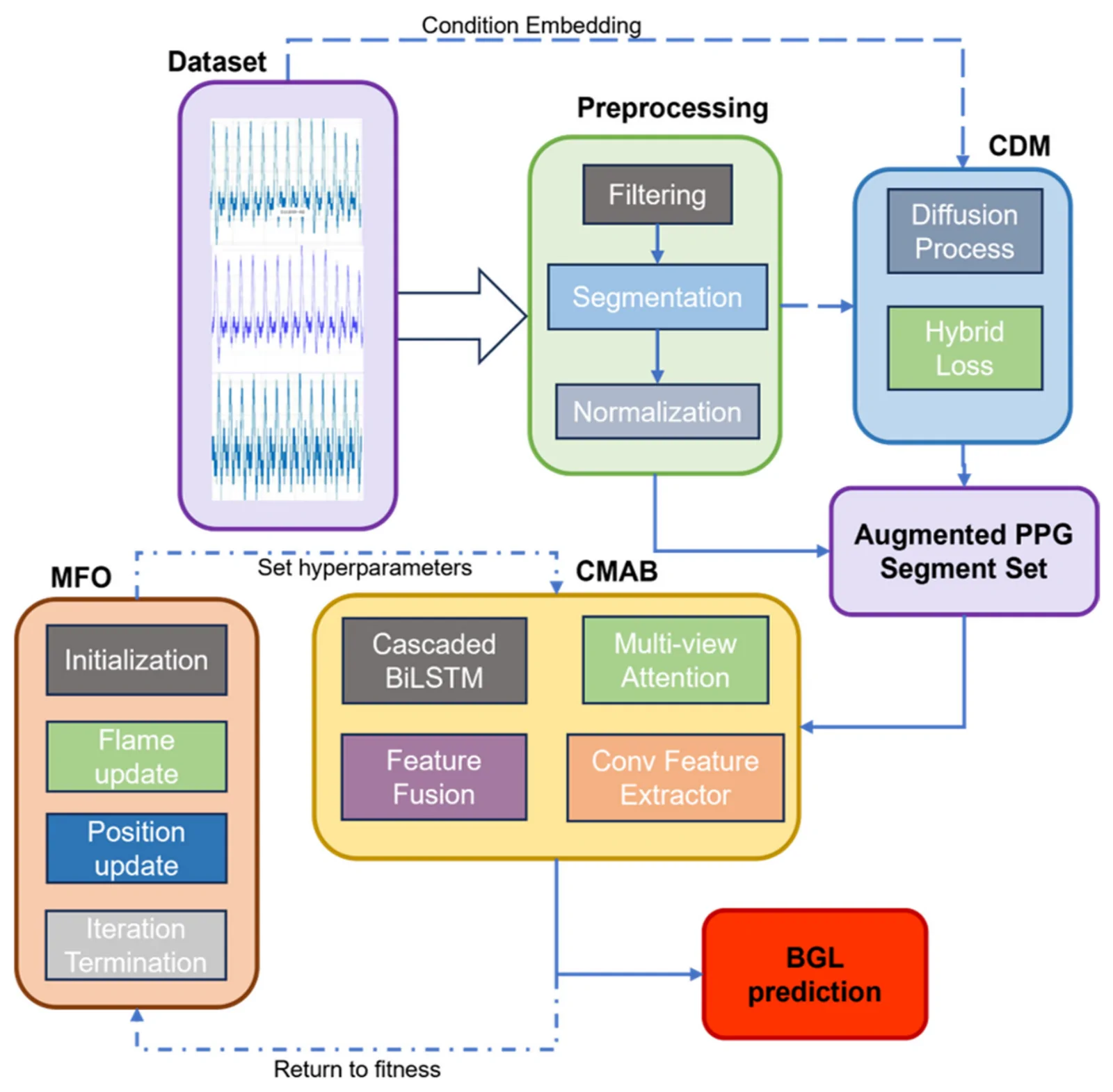
Overall framework of the proposed pipeline. BGL, blood glucose level; CDM, conditional diffusion model; CMAB, cascaded multi-view attention bidirectional long short-term memory network; MFO, moth–flame optimization. After preprocessing, real PPG segments and their BGL, HR, and BMI conditions are used to train the CDM, which generates condition-guided synthetic PPG only within the current outer-training partition. Real and retained synthetic segments are then used to train the CMAB regressor. MFO searches the regression hyperparameters on the subject-wise inner-validation subset: candidate hyperparameters are supplied to regression-model training, and the corresponding inner-validation RMSE is returned as the MFO fitness value. The held-out outer-test fold contains only real PPG and is used once for final evaluation.

The framework proposed in this study is shown in Figure 1 and consists primarily of three core modules: (1) a CDM module; (2) a CMAB network; and (3) an MFO-based hyperparameter optimization module.

First, the raw PPG signal is preprocessed and fed into the CDM together with the corresponding physiological conditions (BGL, HR, and BMI). The CDM generates additional condition-guided PPG waveform realizations around physiological states already represented in the outer-training data; these synthetic segments are used only as augmentation and are not treated as new independent glucose measurements. The real and retained synthetic training segments are then provided to the cascaded multi-view attention BiLSTM network for feature extraction and blood glucose regression. Finally, MFO is used within each outer-training partition to select fold-specific regression hyperparameters. The framework therefore integrates conditional waveform augmentation, temporal feature modeling, and training-only hyperparameter selection.

### 2.2. Dataset

This study used a publicly available PPG dataset developed by the Digital Systems Research Group at the University of Science and Technology of Mazandaran, Behshahr, Iran [20]. The dataset contains measurements from 23 volunteers; participant-level clinical diagnostic status was not reported in the public metadata. The participants comprised 15 males and 8 females, with ages ranging from 22 to 61 years. Demographic and physiological information, including age, sex, height, weight, heart rate, body mass index, and reference blood glucose level, was also provided.

The PPG signals were acquired using an optical sensing system consisting of a green light source with a wavelength of 550 nm and an APDS-9008 photodetector. The signals were sampled at 2175 Hz and digitized using a 12-bit analog-to-digital converter controlled by an ATmega328P microcontroller (Microchip Technology Inc., Chandler, AZ, USA). Hardware filtering was applied during data acquisition, and the resulting signals were stored in MAT and CSV formats. The public dataset was organized into RawData, Labels, and Figures directories.The main characteristics of the dataset are summarized in Table 1.

**Table 1:** Dataset details.

Statistical Items	Value/Description
Total number of participants	23
Number of original PPG recordings	67
Participant-specific recording counts	Not summarized in the public metadata; filenames encode participant ID and within-participant acquisition number
Gender distribution	15 men and 8 women
Clinical diagnosis	Not reported in public metadata
Age range (years)	22~61
Reference BGL, original labels (mg/dL)	88–183
Reference BGL used for modeling (mmol/L)	4.89–10.17
Height (cm)	154~187
Weight (kg)	42~103
Signal sampling frequency (Hz)	2175
Recording duration	674,621 ms raw; cropped to 10 s × 67 (670 s; 21,750 samples/recording)
ADC resolution	12
Optical sensor wavelength (nm)	550
Reference glucose device	Accu-Chek Active self-monitoring blood glucose device; calibration kit 333
Glucose-measurement timing	Measured after PPG acquisition; exact time interval not reported
Blood specimen type	Not reported in public metadata
Repeat measurements/repeatability	Not reported in public metadata

The original blood glucose labels in the public dataset were provided in mg/dL. Before model development, every glucose label was converted using BGL (mmol/L) = BGL (mg/dL)/18, equivalent to 1 mmol/L = 18 mg/dL. Thus, the original range of 88–183 mg/dL corresponded to 4.89–10.17 mmol/L after conversion. This conversion was performed before construction of the CDM conditioning variables and regression targets; all BGL conditions supplied to the CDM, regression targets, model predictions, and RMSE/MAE calculations were therefore expressed in mmol/L throughout model development and evaluation.

A total of 67 original PPG recordings were available from the 23 participants. The raw files had slightly variable durations, with a total recorded duration of 674,621 ms. For model development, each recording was uniformly cropped to a 10 s interval (21,750 samples at 2175 Hz) and was associated with one reference blood glucose measurement. The effective total duration used for modeling was therefore 670 s. The original reference blood glucose values ranged from 88 to 183 mg/dL, corresponding to 4.89–10.17 mmol/L after unit conversion. Each standardized 10 s recording was subsequently divided into two consecutive, non-overlapping 5 s segments, resulting in a total of 134 real PPG segments before data augmentation. The two segments derived from the same original recording inherited the same reference blood glucose label.

According to the public dataset documentation [20], reference blood glucose was measured after PPG acquisition using an Accu-Chek Active self-monitoring blood glucose device with calibration kit 333, and the reported meter value was used as the label for the corresponding recording. The public documentation does not specify the exact time interval between PPG acquisition and glucose measurement, the blood specimen type, whether duplicate or repeated glucose measurements were performed, or within-session measurement repeatability. Because this study is a secondary analysis of the released dataset, these unreported acquisition details could not be reconstructed or verified retrospectively. They are therefore reported explicitly as unavailable rather than inferred and are treated as limitations when interpreting the reference-label precision. The public documentation likewise does not provide physician-confirmed diabetes diagnoses or healthy/type 2 diabetes labels. Accordingly, the clinical composition of the 23-person cohort cannot be determined from the released metadata, and participants were not retrospectively classified as diabetic or non-diabetic from a single reference glucose measurement.

**Figure 2:**
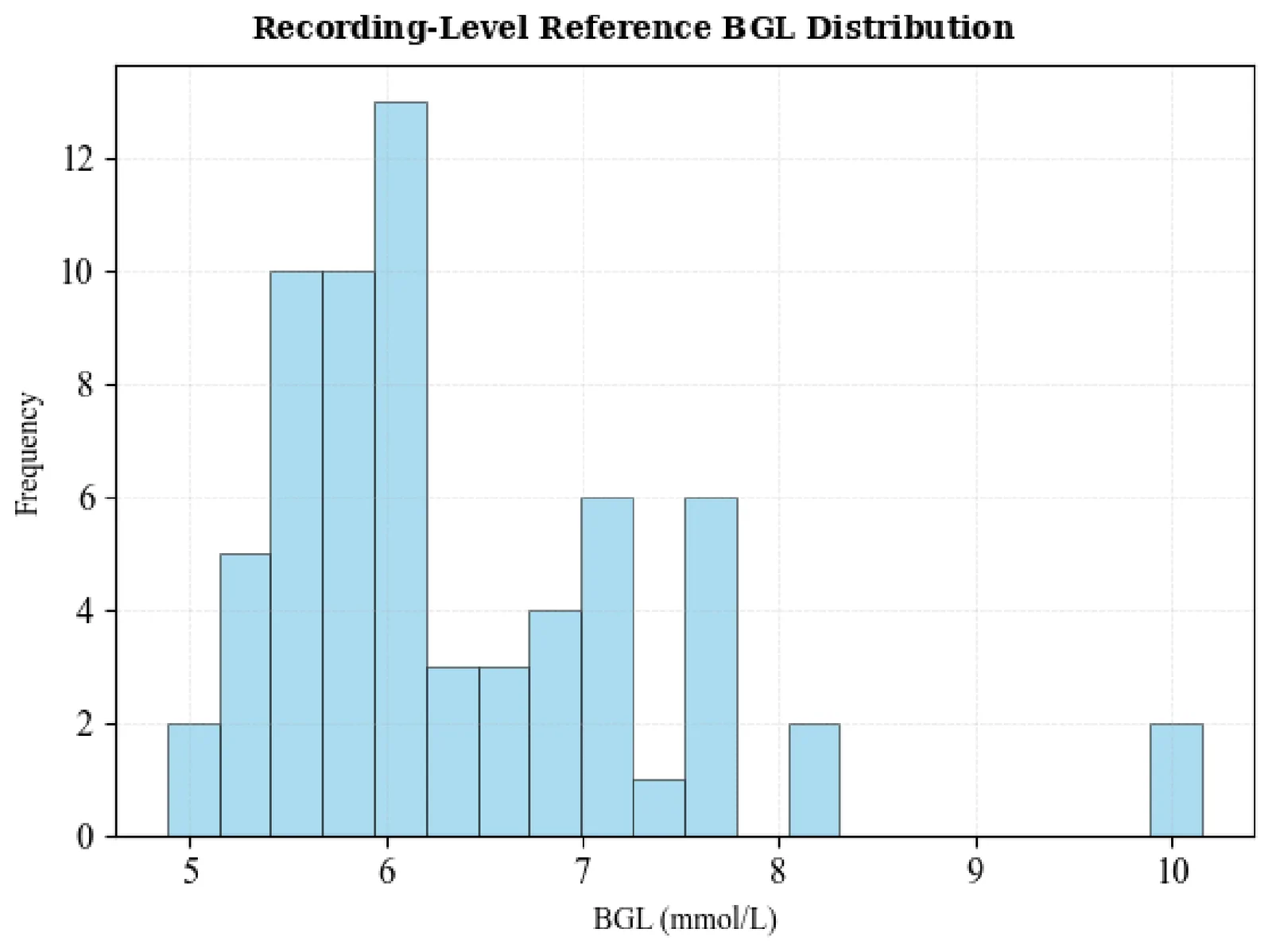
Recording-level reference BGL distribution after unit conversion (n = 67 original 10 s recordings from 23 participants; BGL in mmol/L; range 4.89–10.17 mmol/L). The histogram uses 20 equal-width bins across the observed range (bin width approximately 0.264 mmol/L).

It should be emphasized that the 134 segments were derived from 67 original recordings and therefore should not be regarded as 134 completely independent glucose measurements. To reduce the risk of information leakage, all segments originating from the same participant were assigned to the same dataset partition. Figure 2 shows the recording-level reference BGL distribution after unit conversion, using one value per original 10 s recording (*n* = 67) and spanning 4.89–10.17 mmol/L. The histogram was displayed using 20 equal-width bins across this observed range (approximately 0.264 mmol/L per bin).

### 2.3. Subject-Wise Five-Fold Cross-Validation

To achieve subject-independent evaluation and reduce the risk of information leakage, five-fold cross-validation was performed at the participant level. For the primary augmentation and ablation experiments, the 23 participants were divided into five mutually exclusive folds using a fixed random seed of 42, and this fold assignment was used consistently for those within-study comparisons. In each cross-validation iteration, four folds were used for model development, and the remaining fold was used for testing. This procedure was repeated five times, ensuring that each participant was included in the test set exactly once. Additional participant-level fold assignments were used only for the sensitivity analysis of the final full model, as described in Section 2.8.1.

All original PPG recordings and the corresponding 5 s segments from the same participant were assigned to the same fold. Consequently, PPG segments derived from one participant could not appear simultaneously in the training and test sets. After each raw recording was cropped to the standardized 10 s interval, the 67 recordings were divided into 134 non-overlapping 5 s segments; however, the two segments derived from the same original recording remained within the same fold and shared the same reference blood glucose value.

In each cross-validation iteration, signal-independent preprocessing settings, including the bandpass-filter cutoff frequencies and segment duration, were treated as fixed signal-processing design choices rather than fold-specific model hyperparameters. The 0.30–15.00 Hz passband was established during preliminary preprocessing development from training-signal spectra, without using blood glucose labels, and was fixed before evaluation of the held-out outer folds. The cutoff pair was not re-selected according to outer-test signals or outer-test prediction performance; the same filter was applied unchanged to the training, inner-validation, and outer-test recordings. Each 5 s PPG segment was independently standardized using its own mean and standard deviation; therefore, no dataset-level waveform-normalization statistics were estimated from or transferred between the training and test folds. The physiological conditions used by the CDM were standardized separately using statistics derived only from the data available for the corresponding CDM training stage, as described in Section 2.5.2. All CDM development procedures, including internal validation, final retraining, and synthetic-data generation, were conducted exclusively within the current outer training folds. Synthetic PPG segments were added only to the corresponding training data, whereas the outer test fold contained only real PPG segments.

Moth–flame optimization and regression-model selection were conducted exclusively within the data available in the current outer-training partition. The corresponding outer-test participants were not used for hyperparameter optimization, regression-model selection, or early stopping. After model development within an outer fold, the resulting fold-specific regression model was evaluated only on the corresponding real outer-test fold.

Fold-specific RMSE, MAE, MARD, and Clarke Zone A and A + B proportions were calculated for each held-out fold and retained as secondary descriptive summaries. For the primary evaluation, the two out-of-fold predictions from each original 10 s recording were averaged to obtain one recording-level prediction, and the 67 recording-level predictions from all five held-out folds were pooled before calculating RMSE, MAE, MARD, and Clarke-zone proportions. Thus, the primary metrics are pooled recording-level out-of-fold estimates rather than an unweighted mean of fold-wise segment-level metrics.

Within the four training folds of each outer cross-validation iteration, the outer-training participants were randomly divided at the participant level into an inner-training subset (80%) and an inner-validation subset (20%), with no participant shared between the two subsets. The same subject-wise 80%/20% inner-validation strategy was used for CDM development and MFO fitness evaluation within each outer fold. A provisional CDM was trained on the inner-training subset, and the inner-validation loss was used to identify the optimal training epoch E*. After E* was determined, the provisional model was discarded, the CDM was reinitialized, and a final fold-specific CDM was retrained for exactly E* epochs using all real PPG segments from the four outer training folds. The physiological-condition normalization statistics were recomputed from these four outer training folds before final retraining. This final CDM was then used to generate synthetic samples for the downstream regression model. The outer test fold remained completely inaccessible during diffusion-model development, synthetic-data generation, hyperparameter optimization, model selection, and early stopping.

### 2.4. Preprocessing

Since PPG signals are susceptible to motion artifacts, environmental noise, and individual variability, effective noise reduction is crucial for improving the accuracy of blood glucose estimation [21]. In this study, the following steps were employed for preprocessing the raw PPG signals.

#### 2.4.1. Bandpass Filtering

To remove baseline drift and high-frequency noise, the raw PPG recordings sampled at 2175 Hz were filtered using a Butterworth band-pass design with cutoff frequencies of 0.30 and 15.00 Hz. The filter-order parameter passed to scipy.signal.butter was N = 4, and the filter was represented in second-order-section (SOS) form. Filtering was performed with scipy.signal.sosfiltfilt, i.e., forward and backward application of the SOS filter, which yields a zero-phase response. The SciPy default edge-padding strategy was retained (padtype = ‘odd’; padlen was not manually specified and therefore used the function default), and no additional edge samples were discarded after filtering.

**Figure 3:**
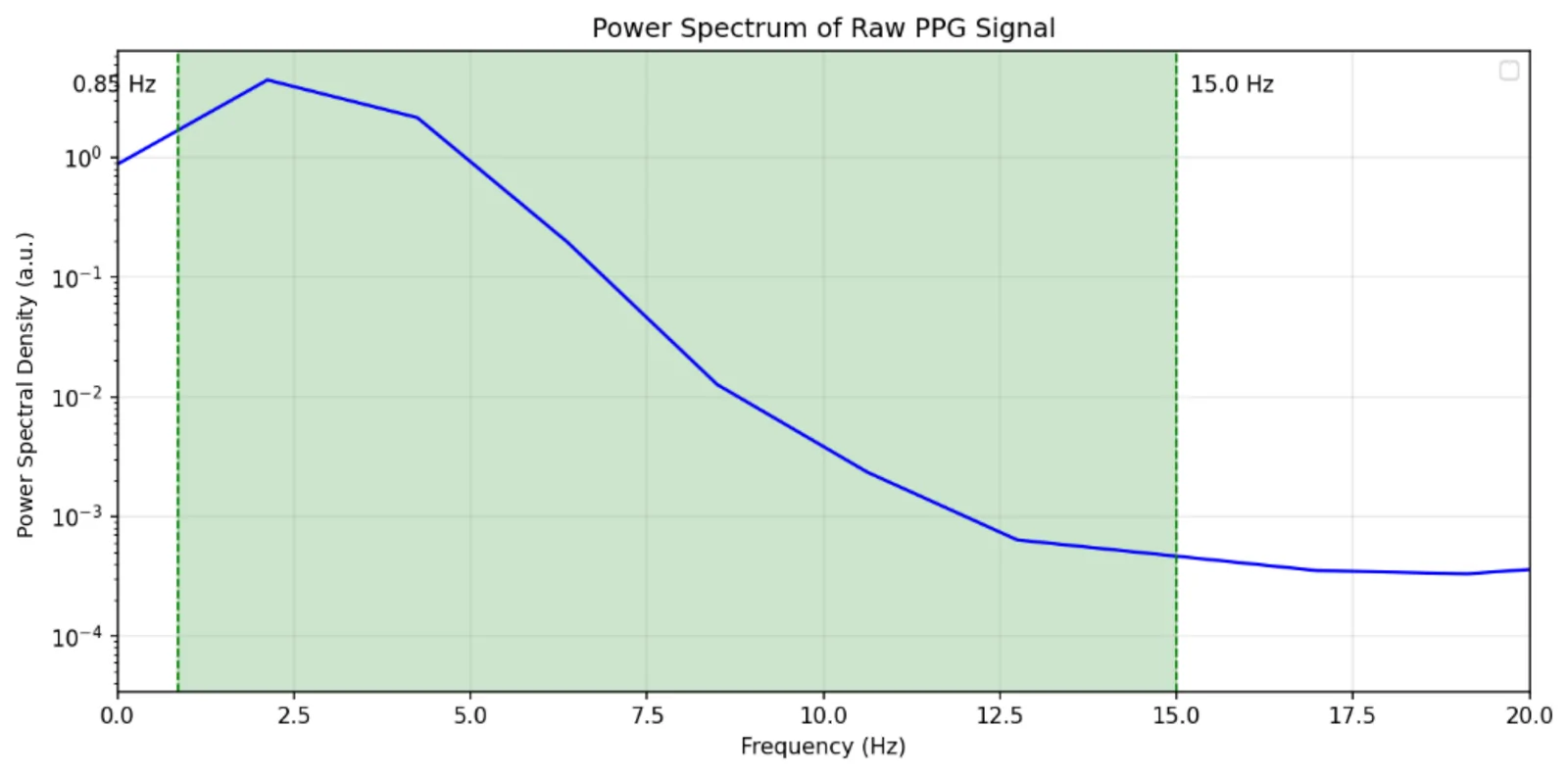
Power spectral density of a representative raw PPG signal from the training data. The ordinate is reported in arbitrary spectral units because the ADC-derived PPG amplitude is not calibrated to an absolute optical-power unit. The dominant pulse-related components were concentrated approximately at 1.06–4.25 Hz, while the shaded 0.30–15.00 Hz interval denotes the intentionally wider fixed preprocessing passband.

**Figure 4:**
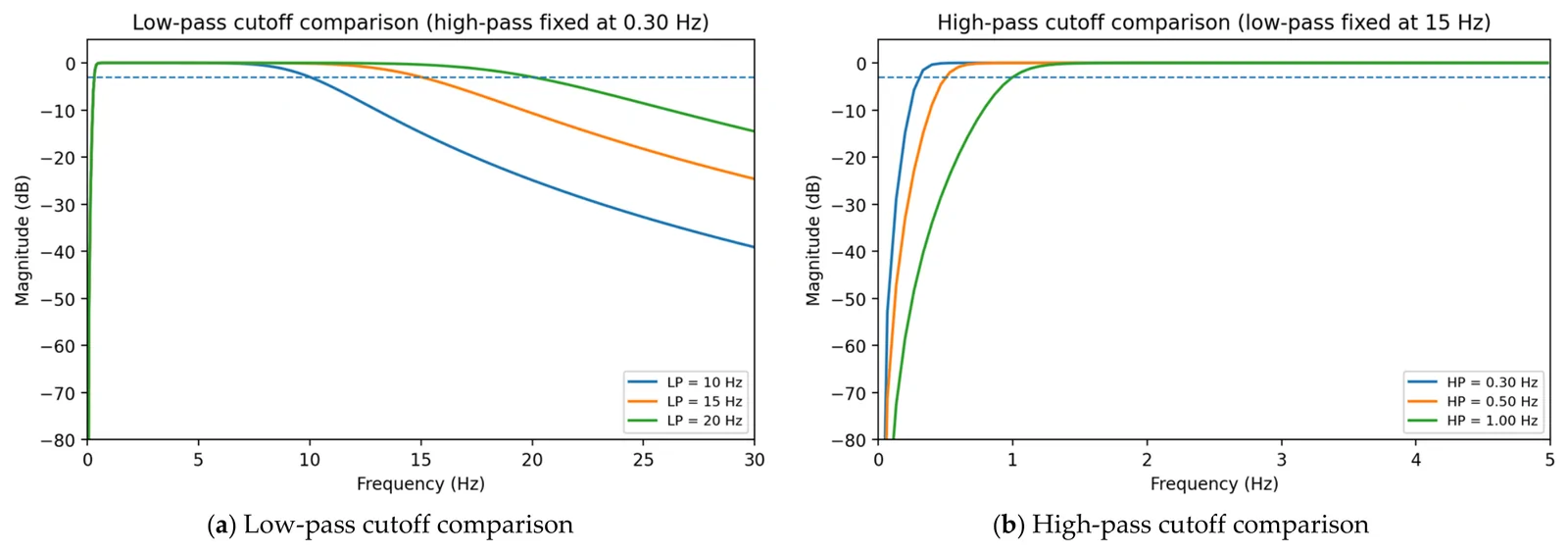
Frequency-response comparison of candidate Butterworth band-pass designs with N = 4. (**a**) Low pass = 10, 15, and 20 Hz, with high pass fixed at 0.30 Hz; (**b**) high pass = 0.30, 0.50, and 1.00 Hz, with low pass fixed at 15 Hz. The dashed line denotes −3 dB.

Inspection of the training-data spectra indicated that the dominant pulse-related components were concentrated approximately between 1.06 and 4.25 Hz. The passband was intentionally kept wider than this dominant spectral interval: the 0.30 Hz high-pass cutoff suppresses very slow baseline drift while retaining slower waveform variation, and the 15 Hz low-pass cutoff preserves pulse morphology and higher-frequency harmonic content while attenuating higher-frequency noise. The cutoff values were selected empirically during preliminary preprocessing development from training-signal spectra without using glucose labels. They were then frozen before outer-fold evaluation and were not adjusted using held-out outer-test signals or model-performance metrics. Figure 3 and Figure 4 are therefore descriptive illustrations of the fixed preprocessing design rather than a fold-specific tuning procedure.

Figure 4 provides a qualitative frequency-response comparison of the candidate cutoff settings. No quantitative signal-to-noise ratio, downstream prediction metric, or outer-test criterion was used to optimize the passband; therefore, the selected 0.30–15.00 Hz band should not be interpreted as a numerically optimized solution. Instead, it was chosen empirically by jointly considering training-data spectra and preservation of pulse-wave morphology. The 0.30 Hz high-pass setting retained more low-frequency waveform content than the 0.50 and 1.00 Hz alternatives, whereas the 15 Hz low-pass setting provided a broader morphology-preserving passband than 10 Hz while attenuating more high-frequency content than 20 Hz. Once selected, this preprocessing configuration remained fixed for all reported cross-validation experiments. The ordinate in Figure 4 represents the digital-filter magnitude response rather than the amplitude of a physiological PPG waveform.

#### 2.4.2. Signal Segmentation

Before segmentation, each raw PPG recording was uniformly cropped to a 10 s interval corresponding to 21,750 samples at 2175 Hz. Each standardized recording was then divided into exactly two consecutive, non-overlapping 5 s segments. These segments cover approximately 5–8 complete cardiac cycles, helping preserve short-term pulse-wave morphology while maintaining a consistent input duration. Each 5 s segment inherited the recording-level reference blood glucose label associated with its original recording, yielding 134 real PPG segments from 67 distinct recording-level glucose measurements contributed by 23 participants.

#### 2.4.3. Normalization

To reduce the influence of segment-specific amplitude and baseline variations, each PPG segment was independently z-score standardized. Specifically, the mean and standard deviation were calculated from the samples within that segment, and the standardized segment was obtained by subtracting its own mean and dividing by its own standard deviation. Because this operation did not use blood glucose labels or statistics from other recordings, it was applied independently to the training, internal-validation, and outer-test segments. This segment-wise normalization avoids transferring dataset-level waveform statistics across participants, but it also removes absolute baseline and amplitude information; consequently, the model primarily learns normalized waveform morphology and relative temporal variation. A separate comparison with a training-derived global waveform-normalization strategy was not performed; therefore, any information loss caused by removal of the absolute amplitude and baseline is treated as a preprocessing limitation rather than assumed to be negligible.

#### 2.4.4. Signal-Quality Handling

The reported preprocessing pipeline did not include an additional signal-quality rejection stage after filtering. All 67 cropped 10 s recordings were retained for downstream segmentation and participant-wise cross-validation, yielding 134 non-overlapping 5 s model inputs. Thus, Figure 3 and Figure 4 were used only to document the spectral characteristics and fixed filter design; they were not used to exclude recordings or adapt preprocessing to held-out participants. Signal morphology analyses reported later in the manuscript were descriptive validation analyses and did not determine sample inclusion or exclusion.

**Figure 5:**
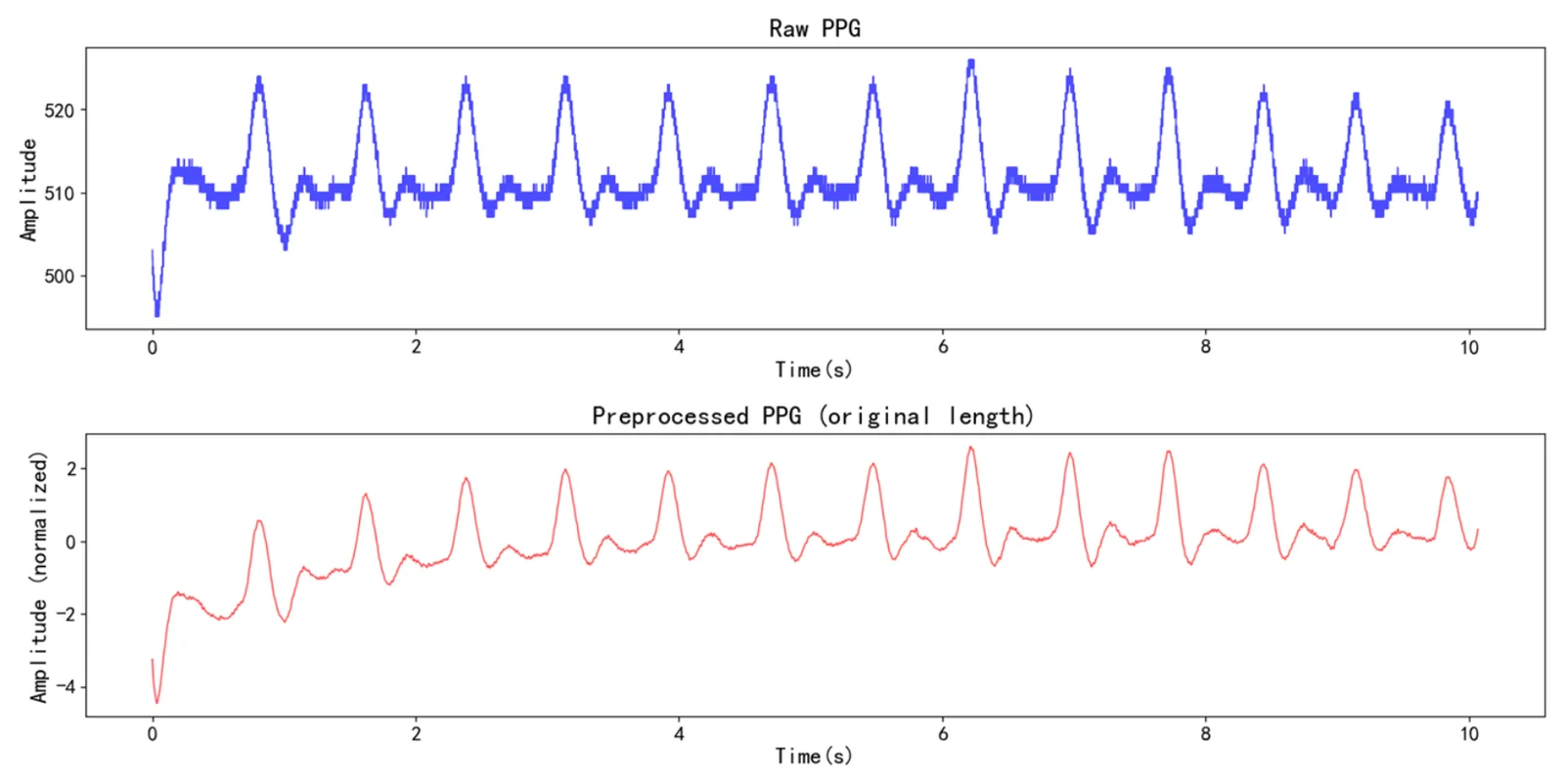
Comparison of a representative raw and preprocessed PPG recording before signal segmentation.

Figure 5 presents a representative full-length PPG recording before and after preprocessing to illustrate the noise-reduction effect. The initial boundary transient visible in the filtered waveform was retained, and no edge samples were manually discarded before the recording was divided into two consecutive non-overlapping 5 s segments for model input. The same deterministic filtering and segmentation procedure was applied to all recordings.

### 2.5. CDM

**Figure 6:**
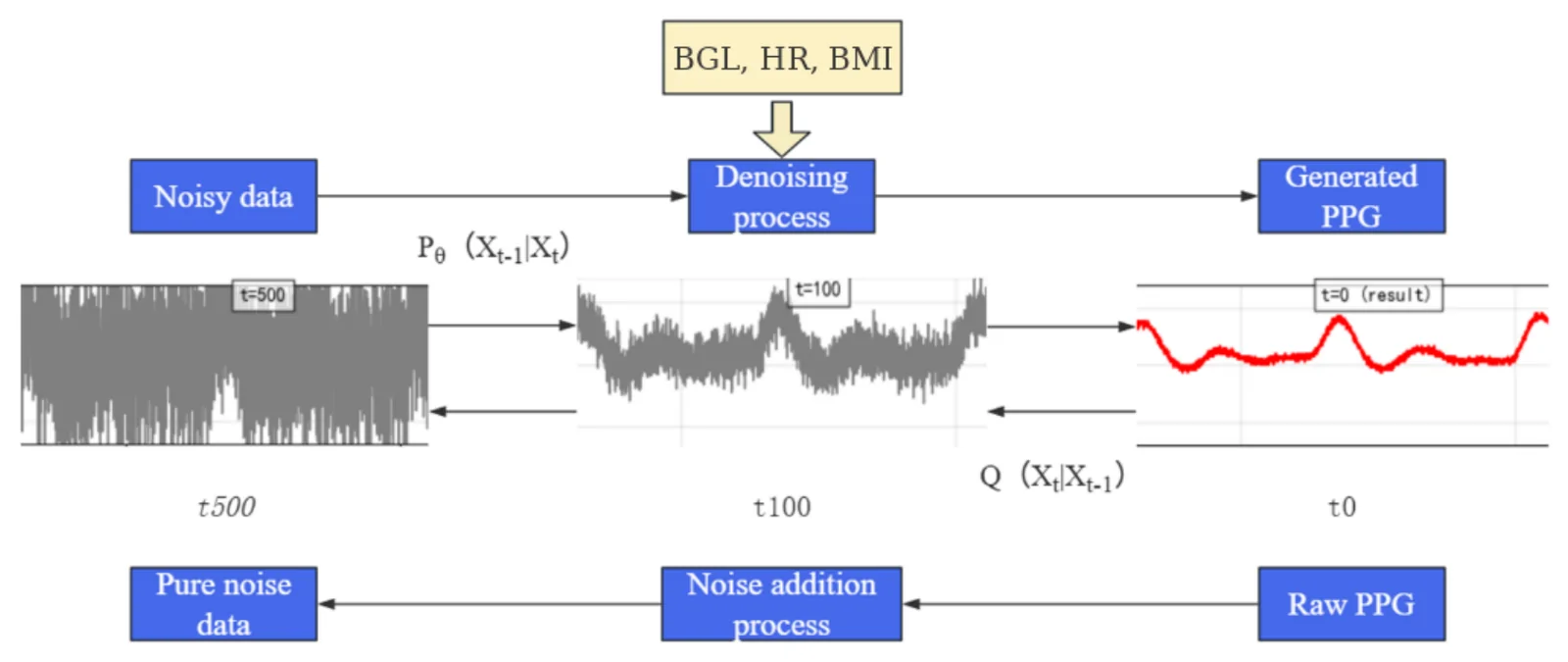
Forward and reverse diffusion processes used by the CDM. In the forward process q(x_t|x_{t − 1}), Gaussian noise is progressively added to the clean PPG x_0 until a highly noisy state is reached. In the learned reverse process p_theta(x_{t − 1}|x_t, C), the denoising network progressively removes noise while conditioning on C = [BGL, HR, BMI] to generate a PPG realization. Here, x_t denotes the PPG state at diffusion step t, theta denotes the learned denoising-network parameters, and T = 1000 diffusion steps were used in the implementation.

Traditional data augmentation methods, such as adding noise, time warping, and scaling, alter waveform morphology but do not explicitly condition the generated variation on the accompanying physiological labels [22,23]. We therefore developed a one-dimensional conditional diffusion model (CDM), following the denoising diffusion probabilistic model formulation [24], to model preprocessed 5 s PPG waveform variation conditional on BGL, heart rate (HR), and body mass index (BMI). During training, Gaussian noise was progressively added to a real PPG segment, and a conditional denoising U-Net was optimized to predict the added noise. During sampling, the learned reverse process generated additional waveform realizations associated with physiological states already represented in the training data. Importantly, conditioning on an observed BGL label does not create new independent glucose information; the generated signals are used as condition-guided augmentation and regularization around existing training labels. The forward and reverse diffusion processes of the CDM are illustrated in Figure 6.

#### 2.5.1. Denoising U-Net Architecture and Diffusion Configuration

The noise-prediction network was implemented as a four-level one-dimensional U-Net with channel dimensions of 64, 128, 256, and 512. The encoder contained three stride-2 downsampling operations followed by a 512-channel bottleneck, and the decoder contained three symmetric upsampling operations. Skip connections linked encoder and decoder features at the same resolution. All one-dimensional convolutional layers used a kernel size of 5. To accommodate input lengths that were not exact powers of two, each decoder feature was interpolated to the temporal length of the corresponding encoder feature before skip-feature fusion. Each resolution block contained two Conv1D layers, with GroupNorm (8 groups) and SiLU (Swish) activation used within the block. An intra-block residual shortcut was used in each residual block, while the same-resolution encoder–decoder connections provided the long U-Net skip pathways. Decoder upsampling was implemented by one-dimensional linear interpolation (mode = ‘linear’) followed by Conv1D. The complete conditional denoising U-Net contained 17,456,512 parameters (approximately 17.46 M).

The forward diffusion process used T = 1000 time steps, with a linear beta schedule increasing from = 1/10,000 to = 0.02. Each diffusion time step was represented using a 128-dimensional sinusoidal embedding and projected to the channel dimension of the corresponding U-Net block. Synthetic PPG segments were generated with the standard DDPM sampler using all 1000 reverse-denoising steps.

#### 2.5.2. Conditional Injection Mechanism

The physiological condition vector was defined as C=[BGL,HR,BMI]. Before embedding, BGL, HR, and BMI were standardized feature-wise. For each CDM training run, the mean and standard deviation of each condition were calculated using only the subjects included in the corresponding CDM training subset, and the same training-derived statistics were then applied to the internal-validation conditions and to the conditions used for synthetic-data generation. No condition-normalization statistics were obtained from the outer test fold. The three standardized scalar conditions were individually projected into 128-dimensional embeddings, forming a three-token physiological representation. At each U-Net resolution level, the current PPG feature representation was projected to 128 dimensions and used as the query, whereas the three condition tokens served as the keys and values in a four-head cross-attention module. The attention output was projected back to the channel dimension of the corresponding U-Net block. The 128-dimensional sinusoidal time-step embedding was separately projected and added to each denoising block. At time step t, the optimization objective of the denoising network $\varepsilon_{\theta }$ is as specified in Equation (1).

At every U-Net resolution level, the four-head physiological cross-attention was implemented as an independent module after each residual block and before the subsequent resolution-changing operation. Accordingly, cross-attention preceded stride-2 downsampling in the encoder and preceded linear-interpolation-based upsampling in the decoder.

(1) $$L_{c}=E_{t,x_{0},\epsilon ,C}\left[{∥\epsilon -\epsilon_{\theta }\left(\sqrt{{\bar{\alpha }}_{t}}x_{0}+\sqrt{1-{\bar{\alpha }}_{t}}\epsilon ,t,C\right)∥}^{2}\right]$$

This conditioning mechanism enables the denoising network to estimate the diffusion noise while preserving compatibility between the generated waveform and the specified BGL, HR, and BMI conditions.

#### 2.5.3. Generation and Expansion

After CDM training, five candidate synthetic PPG segments were generated for each real training segment under the same BGL, HR, and BMI conditions, with independent Gaussian noise used to initialize each DDPM sampling trajectory. In the implemented pipeline, two of these five candidates were then retained by random selection using the fixed seed of 42, producing a real-to-synthetic ratio of 1:2. The five-to-two procedure was an implementation choice for stochastic candidate generation followed by fixed-size subsampling; no quality score or ranking criterion was used, and we do not claim that it is statistically superior to directly generating two independent candidates. The 1:2 ratio was treated as a fixed augmentation setting rather than an empirically optimized hyperparameter, and it was not selected using outer-test performance. Candidate generation and selection were performed independently within each cross-validation training fold; no synthetic sample was generated from or added to an outer-test fold. Because a dedicated augmentation-ratio sensitivity analysis was not performed, the reported augmentation benefit should be interpreted specifically for the 1:2 setting evaluated here.

**Figure 7:**
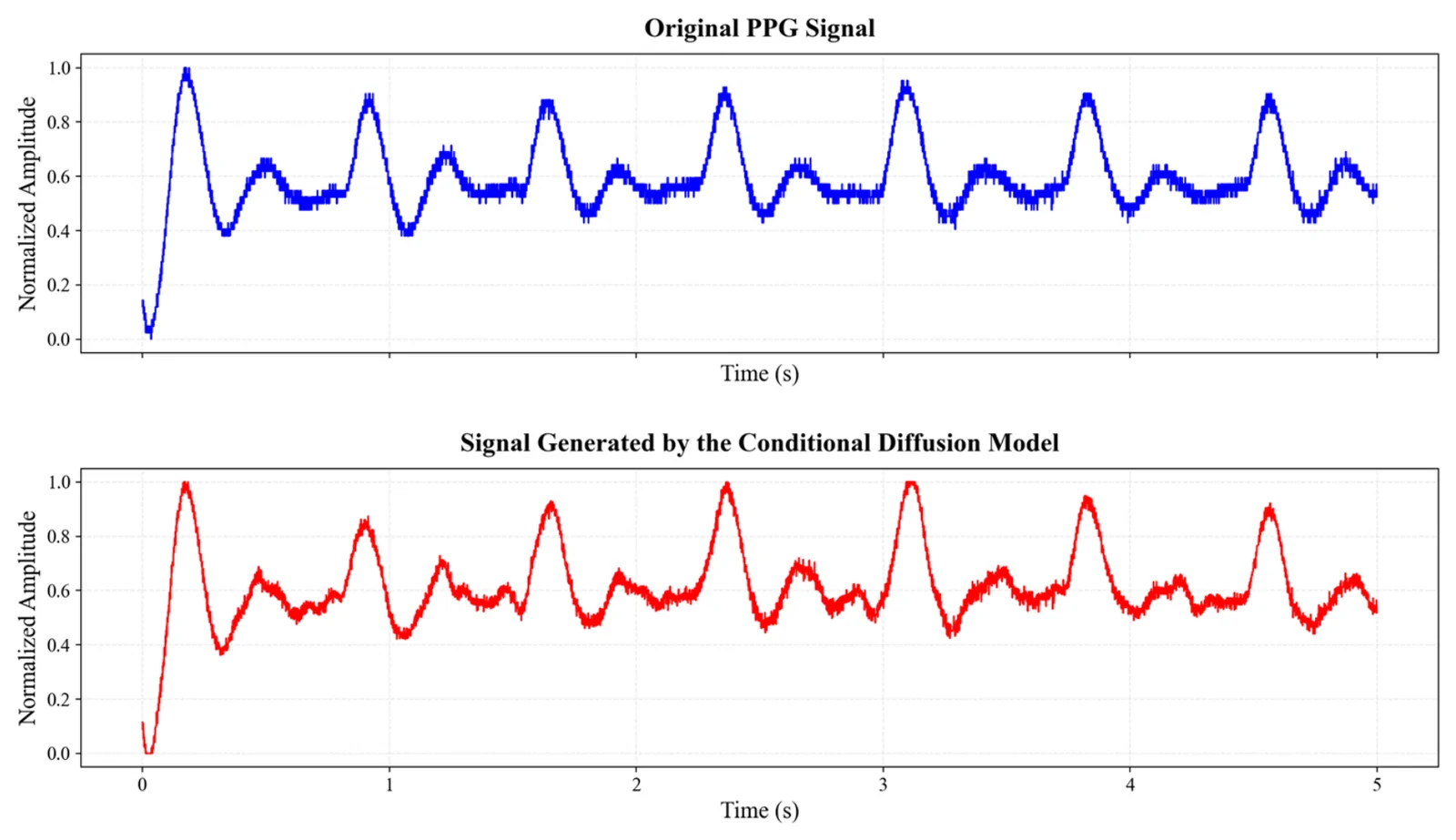
Representative real and generated PPG segments under matched BGL, HR, and BMI conditions. Traces were independently min–max rescaled to [0, 1] for visualization; training used z-score-standardized signals. The exact numeric condition triplet for this original qualitative pair was not retained and is not retrospectively inferred;

As shown in Figure 7, the conditionally generated PPG segment reproduces the periodic pulse pattern and the approximate spacing of dominant peaks observed in the real signal, while retaining secondary waveform components. The two curves are not expected to be pointwise identical because the CDM samples from a conditional distribution rather than reconstructing the input signal. Local differences in amplitude and high-frequency fluctuation remain. A more detailed morphology-based comparison is presented in Section 3.5.

#### 2.5.4. Design of a Hybrid Loss Function

To encourage both accurate noise estimation and preservation of local pulse morphology, a hybrid objective was used. In addition to the mean squared error (MSE) noise-prediction loss, a one-dimensional Structural Similarity Index Measure (SSIM) loss was introduced to compare the original clean PPG segment $x_{0}$ with the clean-signal estimate ${\hat{x}}_{0}$, reconstructed from the predicted diffusion noise [25,26].

At a sampled diffusion step t, the estimated clean PPG signal ${\hat{x}}_{0}$ was reconstructed from the noisy input $x_{t}$ and the predicted noise $\varepsilon_{\theta }\left(x_{t},t,C\right)$, as follows:

(2) $${\hat{x}}_{0}=\frac{\left[x_{t}-\sqrt{1-{\bar{\alpha }}_{t}}\cdot \varepsilon_{\theta }\left(x_{t},t,C\right)\right]}{\sqrt{{\bar{\alpha }}_{t}}}$$

Because the PPG segments were z-score normalized and therefore had no fixed amplitude range, both $x_{0}$ and ${\hat{x}}_{0}$ were clipped to $\left[-3,3\right]$ and linearly rescaled to $\left[0,1\right]$ before SSIM calculation. The rescaled signals were denoted by $x_{0}^{\prime }$ and ${\hat{x}}_{0}^{\prime }$, respectively. The SSIM dynamic range was fixed at 1. The stability constants were set to $C_{1}={\left(0.01\right)}^{2}$ and $C_{2}={\left(0.03\right)}^{2}$, and local statistics were computed using an 11-sample Gaussian window with a standard deviation of 1.5.

For two one-dimensional signals x and y, the SSIM was calculated as follows:

(3) $$SSIM\left(x,y\right)=\frac{\left[\left(2\mu_{x}\mu_{y}+C_{1}\right)\left(2\sigma_{xy}+C_{2}\right)\right]}{\left[\left({\mu_{x}}^{2}+{\mu_{y}}^{2}+C_{1}\right)\left({\sigma_{x}}^{2}+{\sigma_{y}}^{2}+C_{2}\right)\right]}$$

Here, $\mu_{x}$ and $\mu_{y}$ denote the local means of x and y, respectively; $\sigma_{x}$ and $\sigma_{y}$ denote their local standard deviations; $\sigma_{xy}$ denotes their local covariance; and $C_{1}$ and $C_{2}$ are stability constants. In this study, $x={x^{\prime }}_{0}$ represents the rescaled original clean PPG segment, whereas $y={{\hat{x}}^{\prime }}_{0}$ represents the rescaled clean-signal estimate. Accordingly, the structural loss was defined as $L_{SSIM}=1-SSIM({x^{\prime }}_{0},{{\hat{x}}^{\prime }}_{0})$.

The final total loss function is

(4) $$L_{total}=\lambda L_{SSIM}+\left(1-\lambda \right)L_{c}$$

The balance coefficient was fixed at λ=0.3, giving the numerical objective $L_{total}=0.3L_{SSIM}+0.7L_{c}$. Here, $L_{c}$ denotes the mean squared error between the true Gaussian noise and the noise predicted by the conditional U-Net. Thus, the diffusion noise-prediction term contributed 70% of the total objective, and the SSIM-based structural term contributed 30%. The same loss configuration was used in every cross-validation fold without tuning on the outer test data.

The value λ = 0.3 was treated as a fixed loss-weighting design choice so that the standard diffusion noise-prediction objective remained the dominant term (70%) and the SSIM component acted as a secondary structural regularizer (30%). It was not tuned against the outer-test folds and should not be interpreted as an empirically optimal weight. The present experiments evaluate the CDM package as a whole and do not include an isolated MSE-only versus MSE + SSIM ablation; consequently, the independent contribution of the SSIM term cannot be inferred from the current results.

#### 2.5.5. CDM Training Protocol

In each outer cross-validation fold, the participants in the four outer training folds were first divided into a subject-wise inner-training subset and an inner-validation subset. A provisional CDM was initialized from scratch and optimized using Adam with $\beta_{1}=0.9$ and $\beta_{2}=0.999$, a learning rate of $1\times 10^{-4}$, and a batch size of 16. Training was allowed to continue for a maximum of 2000 epochs, with early stopping applied when the inner-validation loss failed to improve for 100 consecutive epochs. The epoch corresponding to the lowest inner-validation loss was recorded as $E^{*}$; the provisional checkpoint was used only to determine $E^{*}$ and was not used for final synthetic-data generation.

After $E^{*}$ was determined, the CDM was reinitialized and retrained for exactly $E^{*}$ epochs using all real PPG segments from the four outer training folds. For this final retraining stage, the means and standard deviations used to normalize BGL, HR, and BMI were recomputed from all subjects in the four outer training folds. The resulting final fold-specific CDM was used to generate synthetic PPG segments for the corresponding augmented training set.

For the primary seed-42 experiments, a random seed of 42 was used for participant-level fold construction, model initialization, mini-batch shuffling, diffusion-noise sampling, and synthetic-candidate selection to improve experimental reproducibility.

To evaluate the contribution of the conditional diffusion augmentation strategy, three training-data configurations were compared under the same subject-wise cross-validation protocol using an identical baseline BiLSTM model: original data only; conventional augmentation using additive noise, time warping, and scaling; and CDM-based augmentation. All model architecture, training, and evaluation settings other than the augmentation strategy were held constant across the three configurations.

For the conventional augmentation baseline, each independently standardized 5 s PPG segment was transformed sequentially using three operations. First, smooth time warping was applied by cubic-spline interpolation with five uniformly distributed control knots and a warping strength of σ = 0.02; stochastic control-point displacements produced a smooth deformation of the temporal axis. Second, the warped segment was multiplied by a global scaling factor sampled independently from U(0.95, 1.05). Third, zero-mean Gaussian white noise was added with a standard deviation equal to 2% of the segment standard deviation [σ_noise = 0.02 × SD(segment)]. Because every 5 s segment had already been z-score standardized, this corresponded to σ_noise = 0.02. Five conventional-augmentation candidates were generated for each real training segment, and two candidates were randomly retained using the fixed seed of 42, yielding a real-to-augmented ratio of 1:2. Thus, the candidate count, random-retention procedure, and final augmentation ratio were matched to the CDM setting so that the comparison differed primarily in the augmentation mechanism rather than in augmentation volume.

### 2.6. CMAB

The CMAB network was developed by adapting the CBHFF architecture proposed in [27], which comprises a convolutional feature extractor, a multi-view attention module, cascaded BiLSTM layers, and a regression head. The principal modifications include replacing the original MLP-based channel-attention branch with a lightweight local cross-channel Conv1D mechanism, replacing the dilated-convolution spatial branch with multi-head temporal self-attention, and introducing coefficient-controlled multiplicative attention fusion. In addition, the outputs of the cascaded BiLSTM layers were projected to a common feature dimension before cross-layer interaction. The cascaded BiLSTM backbone itself is inherited from CBHFF; therefore, the present study evaluates the revised CMAB architecture as an integrated package and does not claim an isolated contribution for the BiLSTM cascade or for any single internal attention/fusion modification.

#### 2.6.1. Convolutional Feature Extractor

To extract translation-invariant local patterns from the preprocessed PPG segments, the front end of the CMAB network employed four one-dimensional convolutional layers for feature encoding.

The output of the lth convolutional layer is represented as

(5) $$F^{\left(l\right)}=ReLU\left(Conv1D\left(F^{\left(l-1\right)},W^{\left(l\right)}\right)+b^{\left(l\right)}\right)$$

The number of filters in the four convolutional layers increases sequentially, ultimately producing a feature map $F\in R^{C\times L}$, where C is the number of channels and L is the time duration.

Specifically, the four Conv1D layers used 64, 128, 256, and 512 output channels. Each convolution used a kernel size of 5, a stride of 1, ‘same’ padding, and a ReLU activation function. No normalization layer was used. Each convolutional layer was followed by a MaxPooling1D layer with a pool size and stride of 2 and a dropout layer with a rate of 0.3.

#### 2.6.2. Multi-View Attention Module

**Figure 8:**
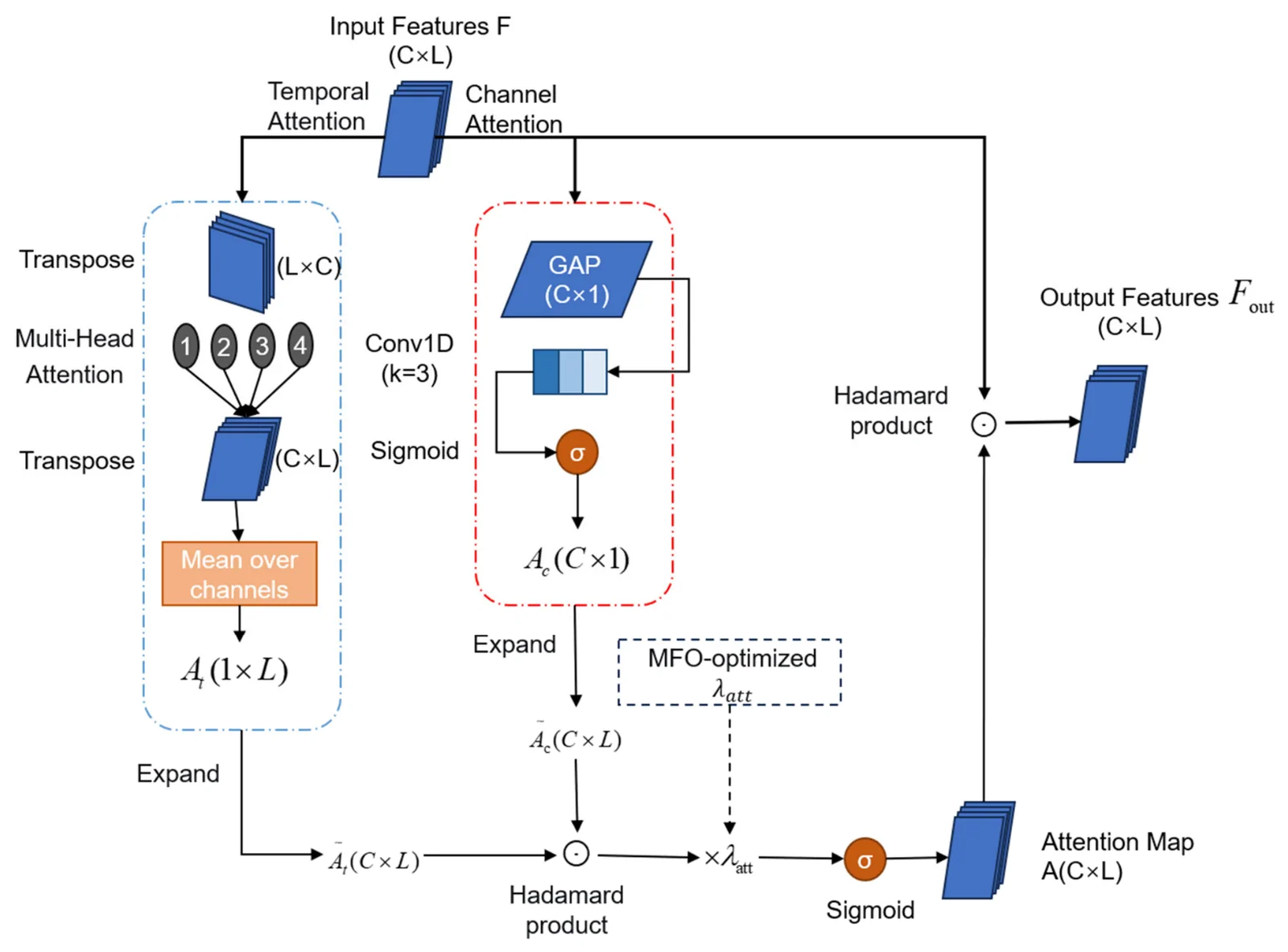
Architecture of the proposed multi-view attention module. Given F ∈ R^C×L^, the channel-attention branch applies temporal global average pooling, one-dimensional convolution (k = 3), and sigmoid activation to obtain A_c ∈ R^C×1^. The temporal-attention branch applies four-head self-attention followed by channel-wise averaging to obtain A_t ∈ R^1×L^, without an additional sigmoid at this stage. After broadcasting both responses to C × L, their Hadamard product is scaled by the MFO-optimized coefficient λ_att and passed through a sigmoid function to obtain the fused attention map A. The output feature map F_out is obtained by element-wise multiplication of A with the original input feature map F. Here, F_gap denotes the temporally pooled channel descriptor, F’_att denotes the output of the temporal self-attention branch after head concatenation and linear projection, Ã_c and Ã_t denote the broadcasted channel and temporal responses, respectively, and ⊙ denotes the Hadamard product. The symbols C and L denote the learned-channel count and temporal length, respectively, while λ_att is the fold-specific MFO-selected fusion coefficient.

To model complementary channel-wise and temporal dependencies in the learned PPG features, we designed a dual-branch multi-view attention module, as illustrated in Figure 8. Given an input feature map F ∈ R^C×L^, where C denotes the number of learned feature channels and L denotes the temporal length, the module constructs channel and temporal responses separately and subsequently fuses them into a joint attention map.

(1) Channel-based attention

The channel-attention branch is designed to assign different weights to learned feature channels. Global average pooling is first performed along the temporal dimension of F to obtain the channel descriptor F_gap ∈ R^C×1^. A one-dimensional convolution with kernel size 3 is then applied along the channel descriptor to model local interactions among neighboring learned feature channels. Finally, a sigmoid function produces the channel-attention map A_c ∈ R^C×1^:

(6) $$A_{c}=\sigma \left(Conv1D\left(F_{gap},k=3\right)\right)$$

where σ(·) denotes the sigmoid function. The one-dimensional convolution provides a parameter-efficient mechanism for local interaction among learned channel descriptors. Importantly, the learned feature channels are not interpreted as explicitly ordered frequency bands; the convolution is used only to capture local dependencies in the learned channel representation.

(2) Temporal attention

The temporal-attention branch models dependencies among temporal positions in the PPG feature sequence. The feature map F ∈ R^C×L^ is first transposed to an L × C sequence and provided to a four-head self-attention module. Queries, keys, and values are derived from the same temporal sequence, and the multi-head attention operation is computed as

(7) $$Attention\left(Q,K,V\right)=softmax\left(\frac{QK^{T}}{\sqrt{d_{K}}}\right)V$$

The outputs of the four attention heads are concatenated and linearly projected, after which the result is transposed back to the C × L arrangement to obtain F′_att. The temporal response A_t is then obtained by averaging F′_att over the channel dimension. No separate sigmoid is applied to A_t before fusion:

(8) $$A_{t}=mean\left({F^{\prime }}_{att},dim=C\right)\in R^{1\times L}$$

Thus, A_t ∈ R^1×L^ summarizes the temporal response learned by self-attention while preserving its pre-fusion response range. The final nonlinear bounding of the joint attention response is performed after the channel and temporal branches are combined. Equation (8) therefore matches the implemented temporal-attention operation: A_t is the channel-wise mean of F′_att and is not passed through a sigmoid before fusion.

(3) Attention fusion

The channel-attention map A_c ∈ R^C×1^ and temporal response A_t ∈ R^1×L^ are broadcast along the temporal and channel dimensions, respectively, yielding Ã_c, Ã_t ∈ R^C×L^. The two broadcasted responses are combined by a Hadamard product. The resulting joint response is scaled by the MFO-optimized attention coefficient λ_att and then passed through a sigmoid function to obtain the final attention map A ∈ R^C×L^:

(9) $$A=\sigma \left(\lambda_{att}\cdot \left({\tilde{A}}_{c}⊙{\tilde{A}}_{t}\right)\right)$$

where ⊙ denotes the Hadamard product and λ_att controls the strength of the joint channel-temporal response. Because A_t is not independently bounded by a sigmoid before fusion, the product Ã_c ⊙ Ã_t is not restricted to [0, 1]. The final sigmoid therefore provides the nonlinear mapping of the fused response to the attention weights. The input features are then reweighted element-wise:

(10) $$F_{out}=F⊙A$$

The coefficient λ_att was treated as a tunable hyperparameter and optimized independently within each outer fold using the MFO procedure described in Section 2.7. Consequently, the pre-sigmoid fusion response is not confined to the narrow range that would arise if both branches had already been bounded to [0, 1]; the final sigmoid alone maps the fused response to the attention-weight range used to reweight F.

#### 2.6.3. Cascaded BiLSTM Layers and Feature Fusion

**Figure 9:**
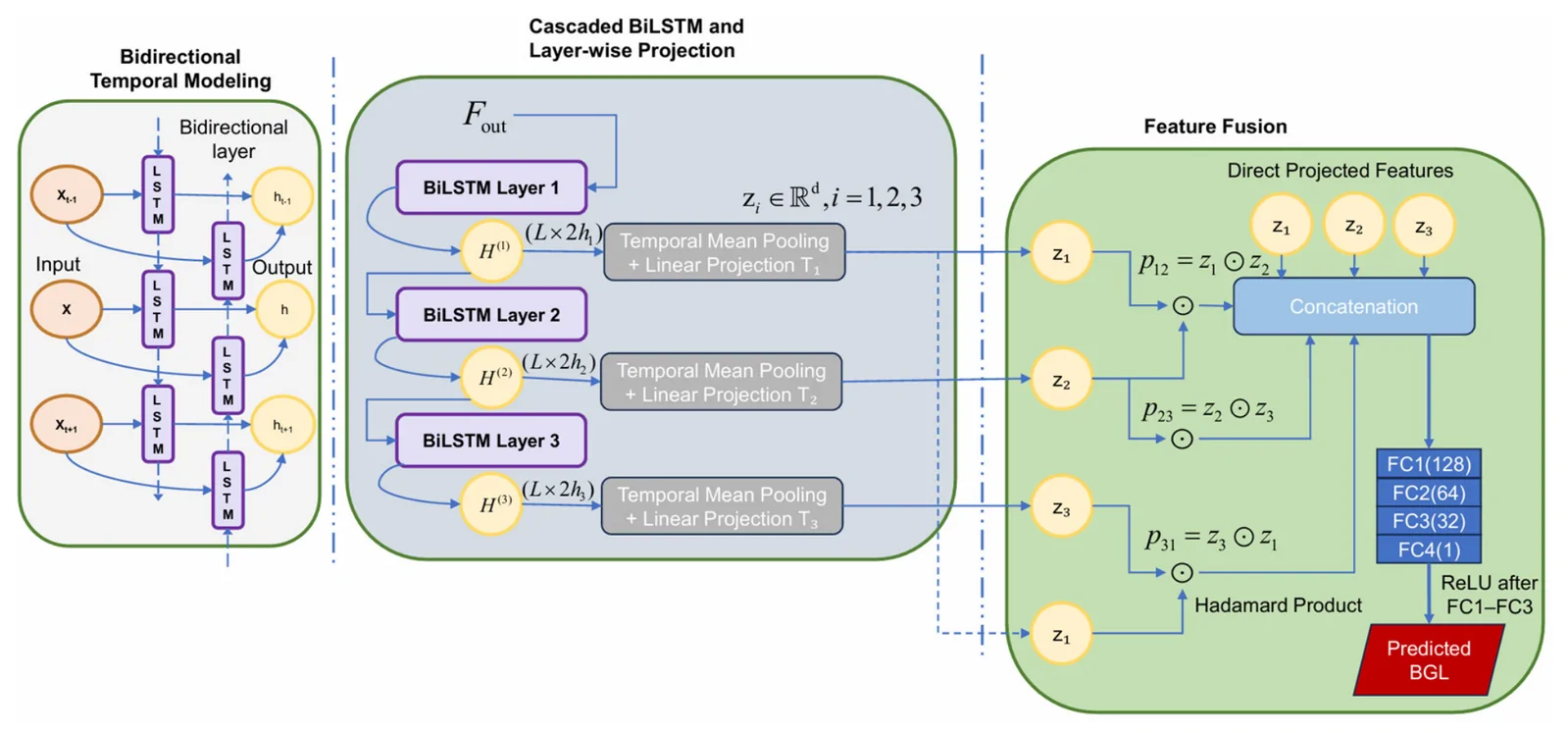
Network architecture of the cascaded BiLSTM and layer-wise feature-fusion regression network. The left panel illustrates bidirectional temporal propagation within a BiLSTM layer. In the central panel, the attention-weighted sequence F_out is processed by three cascaded BiLSTM layers to produce layer-wise output sequences H^(1)^, H^(2)^, and H^(3)^, each with length L and bidirectional feature dimension 2h_i_. Temporal mean pooling followed by an independently parameterized linear projection T_i_ maps each layer output to a fixed-length d-dimensional representation z_i_. In the feature-fusion panel, z_1_, z_2_, and z_3_ are retained as direct projected features and are also used to construct the pairwise interaction features p_12_, p_23_, and p_31_ through Hadamard products. The six vectors are concatenated and passed through the four-layer fully connected regression head to obtain the predicted BGL. Because z_1_, z_2_, and z_3_ are each d-dimensional, p_12_, p_23_, and p_31_ are also d-dimensional, and the concatenated fusion vector F_fusion has dimension 6d.

The attention-weighted feature sequence is processed by a three-layer cascaded BiLSTM network to model bidirectional temporal dependencies at progressively deeper representation levels [27]. The left panel of Figure 9 illustrates the forward and backward propagation within a BiLSTM layer. For a time step t, the forward hidden state and backward hidden state are updated as follows:

(11) $$h_{t}^{\to }=LSTM\left(x_{t},h_{t-1}^{\to }\right),\;h_{t}^{\leftarrow }=LSTM\left(x_{t},h_{t+1}^{\leftarrow }\right)$$

The two directional hidden states are concatenated to obtain the bidirectional representation at the current time step:

(12) $$h_{t}=\left[h_{t}^{\to };h_{t}^{\leftarrow }\right]$$

For layer-wise fusion, the complete temporal output sequence from each BiLSTM layer is retained rather than using only a terminal time step. Let H^(1)^, H^(2)^, and H^(3)^ denote the output sequences of the three cascaded layers. Each H^(i)^ has sequence length L and bidirectional feature dimension 2h_i_, where h_i_ is the hidden-unit count per direction in layer i. Let B_i_ denote the transformation implemented by the i-th BiLSTM layer. The cascaded relation is defined as

(13) $$\begin{array}{c} H^{\left(1\right)}=B_{1}\left(F_{out}\right)\\ H^{\left(2\right)}=B_{2}\left(H^{\left(1\right)}\right)\\ H^{\left(3\right)}=B_{3}\left(H^{\left(2\right)}\right) \end{array}$$

To convert each temporal sequence into a fixed-length layer-level representation, temporal mean pooling is applied across all L time steps. Because the three BiLSTM layers can have different hidden dimensions, the pooled vectors are subsequently mapped by independently parameterized linear projections T_1_, T_2_, and T_3_ to the same d-dimensional space, yielding z_1_, z_2_, and z_3_:

(14) $${\bar{h}}^{\left(i\right)}=\frac{1}{L}\sum_{t=1}^{L}H_{t}^{\left(i\right)},\;\;\;\;\;\;z_{i}=T_{i}\left({\bar{h}}^{\left(i\right)}\right)=W_{i}{\bar{h}}^{\left(i\right)}+b_{i},\;\;\;i=1,2,3$$

The projected representations z_1_, z_2_, and z_3_ are retained directly for fusion and are simultaneously used to construct pairwise cross-layer interaction features through Hadamard products. The three direct projected features and the three interaction features are concatenated to obtain the final fused representation:

(15) $$\begin{array}{c} p_{12}\;=\;z_{1}⊙z_{2},\;\;\;\;p_{23}\;=\;z_{2}⊙z_{3},\;\;\;\;p_{31}\;=\;z_{3}⊙z_{1}\\ F_{fusion}\;=\;\left[z_{1};z_{2};z_{3};p_{12};p_{23};p_{31}\right] \end{array}$$

As shown in Figure 9, temporal mean pooling explicitly converts each BiLSTM output sequence into a fixed-length representation using information from all time steps rather than relying on a single terminal hidden state. Independent projections align the three layer-level representations to the common dimension d, which permits element-wise cross-layer interactions. The concatenated fusion vector therefore contains both the direct projected features and their pairwise interaction terms and has dimension 6d. This vector is passed to a four-layer fully connected regression head. The first three fully connected layers use the ReLU activation function, denoted by φ(·), whereas the final layer directly outputs the estimated blood glucose level:

(16) $$\hat{y}=W_{4}\phi (W_{3}\phi {(W}_{2}\phi {(W}_{1}F_{fusion}+b_{1})+b_{2})+b_{3})+b_{4}$$

The hidden-unit counts h_1_, h_2_, and h_3_ of the three BiLSTM layers were treated as tunable hyperparameters and selected independently within each outer fold using the ranges specified in Section 2.7; consequently, the bidirectional output dimension of layer i was 2h_i_. The three layer-level vectors were independently projected to the same dimension d before Hadamard interaction and concatenation. The regression head consisted of four fully connected layers with output dimensions of 128, 64, 32, and 1. ReLU activation was applied after FC1-FC3, while the dropout rate was selected as a fold-specific hyperparameter.

#### 2.6.4. Model Training Configuration

The regression network was trained using mean squared error loss and the Adam optimizer. In each outer fold, the learning rate, the three BiLSTM hidden-unit counts, the dropout rate, and the attention coefficient were set to the fold-specific values selected by MFO. For model-selection purposes, a provisional regression model was trained on the 80% subject-wise inner-training subset and monitored on the 20% inner-validation subset. The batch size was fixed at 16, the maximum number of training epochs was 300, and early stopping was triggered when the inner-validation loss failed to improve for 30 consecutive epochs. The epoch corresponding to the lowest inner-validation loss was recorded as E_reg*. After E_reg* had been determined, the provisional regression model was discarded, the model was reinitialized with the fold-specific MFO-selected hyperparameters, and the final regression model was retrained for exactly E_reg* epochs using the augmented data from all participants in the four outer training folds. The resulting model was then evaluated once on the corresponding real outer-test fold. Thus, outer-test participants were not used for hyperparameter optimization, epoch selection, early stopping, or model fitting.

### 2.7. Hyperparameter Tuning Based on the MFO

To automate hyperparameter selection for the BiLSTM regression model, this study employed moth–flame optimization (MFO) [28,29], a population-based metaheuristic in which candidate solutions are updated relative to adaptively selected flames. MFO was used here as an automated search strategy rather than assumed a priori to be superior to other optimizers. Because particle swarm optimization and genetic algorithms were not evaluated in this study, no claim is made that MFO converges faster or avoids local optima more effectively than those methods. The optimized variables were the learning rate, the hidden-unit counts of the three BiLSTM layers, the dropout rate, and the attention coefficient, with validation RMSE used as the fitness criterion. A matched-budget Random Search comparison was additionally performed to distinguish the effect of MFO from that of generic hyperparameter optimization.

#### 2.7.1. Optimizing Variable Definitions

Encode the hyperparameters to be optimized as ‘moth’ position vectors in the MFO algorithm:

(17) $$X=\left[lr,n_{h_{1}},n_{h_{2}},n_{h_{3}},d_{rate},\lambda_{att}\right]$$

where

Learning rate (lr): 10^−4^ to 10^−2^; searched in log-space (log-uniform).

Hidden units (h1, h2, h3): Layer 1, 4–16; Layer 2, 8–20; Layer 3, 8–24. Continuous MFO positions were mapped to integer hidden-unit counts using np.rint(), converted to integers, and clipped to the predefined bounds.

Dropout rate (d_rate): 0.2–0.5.

Attention coefficient (lambda_att): 0.1–0.8.

Fitness function: validation RMSE at the 5 s window level (mmol/L). Within each outer-training partition, participants were randomly divided subject-wise into 80% inner-training and 20% inner-validation subsets. MFO fitness was calculated only on the inner-validation participants, and the outer-test participants were not used for fitness evaluation or hyperparameter selection.

#### 2.7.2. MFO Optimization Process

Initialization and reproducibility settings: MFO was run independently within each outer fold using 10 moths and a fixed MFO random seed of 42. Each moth represented one candidate hyperparameter configuration.

Flame-count update: The number of active flames was dynamically adjusted according to Equation (18):

(18) $$F_{l}=round\left(N-l\cdot \frac{N-1}{T}\right)$$

where l is the current iteration number, N = 10 is the moth population size, and T = 20 is the maximum number of MFO iterations.

Moth-position update: Each moth moved toward the corresponding flame along the logarithmic spiral path defined in Equation (19).

(19) $$X_{i}^{t+1}=D_{i}e^{br}cos\left(2\pi r\right)+F_{j}^{t},\;D_{i}=\left|F_{j}^{t}-X_{i}^{t}\right|$$

The spiral constant b was set to 1. Candidate positions falling outside the predefined search ranges were clipped to the corresponding lower or upper bounds; hidden-unit variables were subsequently rounded using np.rint(), converted to integers, and clipped again to their valid integer ranges.

#### 2.7.3. Iteration Termination

The MFO search was terminated when either the maximum of 20 iterations was reached or the best validation RMSE failed to improve for five consecutive iterations.

With 10 moths and a maximum of 20 iterations, the maximum nominal search budget was 200 candidate evaluations per outer fold. Because of early stopping, the actual numbers of candidate evaluations were 160, 190, 200, 150, and 180 for Outer Folds 1–5, respectively (880 evaluations in total).

MFO was executed independently on the participant-wise inner-training and inner-validation data of every outer fold. Consequently, each outer fold yielded its own selected combination of learning rate, BiLSTM hidden-unit counts, dropout rate, and attention coefficient. The fold-specific configurations are reported in Table 2. No single globally optimized hyperparameter vector was reused across the five outer folds. Importantly, MFO fitness and hyperparameter selection were based exclusively on the corresponding inner-validation participants; neither the outer-test participants nor the outer-test RMSE were used for hyperparameter selection or model selection.

#### 2.7.4. Matched-Budget Random Search Comparison

To evaluate whether the observed benefit was specific to MFO rather than to hyperparameter tuning in general, Random Search was implemented as a standard comparison under a matched evaluation budget. Random Search used the same participant-wise inner training/validation partitions, model architecture, training procedure, fitness function, and hyperparameter search ranges as MFO. The learning rate was sampled log-uniformly from 10^−4^ to 10^−2^; the three hidden-unit counts were sampled as discrete integers from 4–16, 8–20, and 8–24; and dropout and the attention coefficient were sampled uniformly from 0.2–0.5 and 0.1–0.8, respectively. To match the actual computational search budget used by MFO after early stopping, Random Search evaluated 160, 190, 200, 150, and 180 candidate configurations in Outer Folds 1–5, respectively. The configuration with the lowest inner-validation RMSE in each fold was then used for final training and evaluation on the same real outer-test participants. This matched-budget analysis is interpreted as a controlled within-study benchmark of the tuning strategy rather than as evidence that MFO is generally superior to Random Search or to other hyperparameter optimizers.

### 2.8. Evaluation Metrics and Statistical Analysis

#### 2.8.1. Experimental Setup

All experiments were conducted using Python 3.9.21 on an NVIDIA GeForce RTX 4060 Laptop GPU. The implementation used PyTorch 2.6.0+cu126 (CUDA 12.6), TensorFlow 2.19.0, and NumPy 1.26.4.

The conditional denoising U-Net contained 17,456,512 trainable parameters (approximately 17.46 M). The diffusion sampler used all 1000 reverse-denoising steps, and the fold-specific MFO searches evaluated 160, 190, 200, 150, and 180 candidate configurations in Outer Folds 1–5, respectively. Wall-clock training time, synthetic-generation time, regression inference time, and peak GPU-memory usage were not prospectively recorded under a fixed profiling protocol during the original experiments; therefore, these quantities are not retrospectively reported as benchmark values. The present implementation should consequently be interpreted as an offline methodological evaluation, and dedicated latency, memory, and energy profiling on target hardware will be required before any wearable or edge-deployment claim can be made.

All experiments were evaluated using subject-wise five-fold cross-validation. The 23 participants were divided into five mutually exclusive folds using the fixed random seed of 42, with four folds used for model development and one fold used for testing in each iteration. All recordings and PPG segments belonging to the same participant were retained within the same fold, and the same fold assignment was used across all experimental comparisons. Each PPG waveform was independently z-score standardized at the segment level, whereas BGL, HR, and BMI used as CDM conditions were standardized using statistics derived exclusively from the corresponding CDM training data.

The original dataset contained 67 ten-second PPG recordings, which were divided into 134 non-overlapping five-second segments. Within each outer-training partition, participants were randomly divided subject-wise into 80% inner-training and 20% inner-validation subsets. A provisional CDM was trained on the inner-training subset and evaluated on the inner-validation subset to determine the optimal epoch E*. The CDM was then reinitialized and retrained for E* epochs using all real segments from the four outer training folds before synthetic-data generation. Five synthetic candidates were generated per real training segment, and two candidates were retained to construct a real-to-synthetic ratio of 1:2. MFO was run independently within each outer fold using the same subject-wise inner-validation strategy to select fold-specific regression hyperparameters. A provisional regression model then used the inner-validation loss with a patience of 30 epochs to determine E_reg*; after model selection, it was discarded, and a final regression model was reinitialized and trained for exactly E_reg* epochs on the augmented data from all four outer training folds. The outer test fold contained exclusively real PPG segments and was not involved in internal validation, data generation, diffusion-model training, regression-model training, early stopping, or hyperparameter optimization. 

The model was independently trained and evaluated in each of the five outer cross-validation iterations. RMSE, MAE, MARD, and the Clarke Zone A and Zone A + B proportions were calculated for each held-out fold and retained as secondary descriptive summaries of fold-wise performance. Because the five outer folds have overlapping training populations and contain different numbers of recording-level observations, the mean and sample standard deviation across the five folds were not used as the primary estimate of predictive uncertainty.

For the primary evaluation, the two out-of-fold predictions obtained from the two non-overlapping 5 s segments of each original 10 s recording were averaged to obtain a single recording-level prediction. Accordingly, the primary evaluation comprised 67 recording-level predictions matched to 67 recording-level reference glucose measurements from 23 participants. These 67 measurement instances are distinct at the recording level, but they were not treated as mutually independent for inferential purposes because some participants contributed multiple recordings. The fold-wise results used for augmentation and ablation comparisons were retained as secondary segment-level analyses because these experiments compare model variants under the same 5 s input unit and identical subject-wise data partitions; these segment-level results are not interpreted as independent glucose measurements. Because the public dataset provides reference glucose values at the recording level rather than a single participant-level target, repeated recordings from the same participant were not collapsed into one participant-level glucose prediction. Such averaging would create a participant-level target not supplied by the dataset and would discard recording-level information. Instead, participant was used as the clustering/resampling unit in the uncertainty analyses described in Section 2.8.8. For pooled out-of-fold performance, the recording-level predictions from all five outer test folds were concatenated, and RMSE, MAE, MARD, and Clarke-zone proportions were computed once on this pooled set; these primary metrics were not obtained by taking an unweighted average of the five fold-specific metrics.

To assess the sensitivity of the final model to a specific participant partition, the complete subject-wise five-fold evaluation of the full model was additionally repeated using two distinct participant-level fold assignments generated with random seeds 2026 and 3407. For each repeated evaluation, all recordings and derived segments from the same participant remained within the same fold, and CDM training, synthetic-data generation, model selection, and regression-model training were restricted to the corresponding outer-training participants. The original seed-42 partition was retained for the primary augmentation and ablation experiments, whereas the additional partitions were used only as a sensitivity analysis of the final full model. For each partition scheme, the primary performance metrics were calculated at the recording level after averaging the two 5 s predictions belonging to each original 10 s recording. This repeated-partition analysis was intended to assess sensitivity to participant-fold assignment and is interpreted descriptively rather than as a formal confidence interval for all sources of training stochasticity.

#### 2.8.2. Evaluation Criteria

Prediction performance was evaluated using three numerical error metrics (RMSE, MAE, and MARD) and the Clarke error grid (CEG) as a geometric error-grid descriptor. The CEG results characterize where reference–prediction pairs fall relative to the standard Clarke zones and are not used here as stand-alone evidence of clinical utility or safety.

(1) RMSE

RMSE is used to measure the deviation between predicted and actual values. It is sensitive to large errors and effectively reflects the model’s predictive stability. Its formula is

(20) $$RMSE=\sqrt{\frac{1}{N}\sum_{i=1}^{N}{\left(y_{i}-{\hat{y}}_{i}\right)}^{2}}$$

where N represents the total number of samples, $y_{i}$ represents the true blood glucose value of the i-th sample, and ${\hat{y}}_{i}$ represents the corresponding model prediction. The units of RMSE are the same as those of blood glucose (mmol/L); a smaller value indicates higher prediction accuracy.

(2) MAE

MAE directly reflects the average magnitude of prediction errors and penalizes large errors more leniently than RMSE. It is calculated as follows:

(21) $$MAE=\frac{1}{N}\sum_{i=1}^{N}\left|y_{i}-{\hat{y}}_{i}\right|$$

The symbols in the formula have the same meanings as above. The unit of MAE is mmol/L; a smaller value indicates a more accurate prediction.

(3) MARD

MARD is one of the most critical evaluation metrics in the field of blood glucose monitoring, used to measure the proportion of deviation between predicted values and true values. Its calculation formula is

(22) $$MARD=\frac{1}{N}\sum_{i=1}^{N}\frac{\left|y_{i}-{\hat{y}}_{i}\right|}{y_{i}}\times 100\%$$

MARD is expressed as a percentage, which eliminates the influence of absolute blood glucose levels on error assessment and facilitates performance comparisons across different blood glucose levels.

(4) CEG

The Clarke error grid is a geometric framework for categorizing reference–prediction pairs according to five predefined zones (A–E) that reflect different error consequences in the original Clarke formulation [30]. In this study, the standard Clarke zone definitions were used as follows:

Zone A: For reference glucose concentrations ≥ 3.9 mmol/L (70 mg/dL), predicted values are within ±20% of the reference value. For reference glucose concentrations < 3.9 mmol/L, both the reference and predicted values are <3.9 mmol/L. Under the original Clarke definition, these reference–prediction pairs fall within Zone A [30].

Zone B: Predicted deviations exceed the range of Zone A but do not lead to inappropriate clinical decisions; these are considered benign errors.

Zones C, D, and E: These are risk zones that may lead to overtreatment or delayed treatment.

Zone assignment was performed programmatically from the reference–prediction pairs using the standard Clarke zone boundaries described in [30]. Code implementing the Clarke zone assignment used in this study is included in Supplementary File S1. The proportions of predictions in the Clarke zones are reported as error-grid agreement descriptors. A higher proportion in Zone A indicates that a larger share of predictions falls within the predefined Zone A limits; however, on the restricted glucose range and small cohort evaluated here, this proportion should not be interpreted by itself as evidence of clinical utility or safety.

#### 2.8.3. Morphological Assessment of Generated PPG Signals

To further assess the morphological fidelity of the generated PPG signals, representative real and generated 5 s segments were compared in terms of waveform structure, peak–valley organization, rise time, and fall time. For visualization and morphological analysis, each segment was independently rescaled to [0, 1], whereas the z-score-normalized signals were retained for model training. Peaks were detected using scipy.signal.find_peaks, with an adaptive amplitude threshold height = max(0.1, 0.5 × SD(signal)) and a minimum inter-peak distance of int(0.25 × fs) = 543 samples (0.25 s at fs = 2175 Hz). Valleys were defined as the minimum-amplitude sample between each pair of adjacent detected peaks; when samples preceded the first detected peak, the minimum in that leading interval was retained as the initial valley. Exactly the same detector settings and valley rule were applied to the real and generated signals. Rise time was defined as the interval from a detected valley to the subsequent peak, whereas fall time was defined as the interval from a detected peak to the subsequent valley. For the representative real and generated signals used in the subsequent morphology analysis, this procedure yielded 14 rise-time intervals and 13 fall-time intervals for the real signal and the same counts for the generated signal; these interval-level observations were used only for descriptive waveform-morphology comparison. 

#### 2.8.4. Evaluation Protocol for Heart-Rate Conditional Consistency

Because heart rate (HR) was explicitly supplied as a physiological condition to the CDM, a quantitative analysis was performed to examine whether this condition was reflected in the generated waveforms. For the 23 generated PPG segments included in this analysis, pulse peaks were detected, and the mean peak-to-peak interval was converted to an estimated HR in beats per minute. The estimated HR was then compared with the corresponding conditioning HR using Pearson’s correlation coefficient, mean absolute error (MAE), and root mean square error (RMSE). This analysis was intended to evaluate the HR-conditional responsiveness of the generator rather than to establish complete physiological validity of the generated signals.

#### 2.8.5. Evaluation Protocol for BGL Conditional Responsiveness

To evaluate whether the CDM responded to the BGL condition independently of HR and BMI, HR and BMI were fixed at 91.38 bpm and 21.45 kg/m^2^, respectively, while the BGL condition was varied across five representative levels within the observed data range (5.43, 5.72, 6.12, 6.89, and 7.56 mmol/L). For each BGL level, 100 PPG segments were generated, yielding 500 synthetic samples in total. A baseline BiLSTM regression model trained exclusively on real PPG data was used as a probe to estimate BGL from the generated signals. For each BGL condition, the probe-estimated values were summarized as mean ± SD. Across all generated samples, Pearson correlation, Spearman rank correlation, and MAE were calculated between the conditioning BGL and the probe-estimated BGL. This analysis was designed to assess BGL-conditional responsiveness of the generated waveforms rather than to establish physiological causality. Because this probe was trained only on real PPG and was never exposed to generated waveforms, the analysis was designed to test whether BGL-conditioned changes in the synthetic signals aligned with the predictive structure learned from real PPG rather than being detectable only through a model adapted to synthetic data. This test reduces, but cannot eliminate, the possibility of generator-specific label artifacts.

#### 2.8.6. Same-Condition Diversity and Distribution-Level Validation of Generated PPG Signals

To further quantify whether the CDM generated condition-consistent yet non-identical waveforms and whether the generated signals preserved the distributional characteristics of real PPG, an additional quantitative validation was performed. For each matched physiological condition, multiple PPG realizations were generated from independent Gaussian-noise initializations. Same-condition diversity was quantified using pairwise Pearson correlation and the normalized Euclidean distance D(x_i,x_j) = ||x_i − x_j||_2/√L, where L denotes the signal length. Higher correlation indicates preservation of the common waveform structure, whereas a non-zero normalized distance indicates that the generated realizations are not identical copies.

For distribution-level comparison, four physiologically interpretable waveform features were extracted from matched real and generated PPG signals: dominant frequency, mean peak interval, rise time, and fall time. Agreement between real and generated features was summarized using the Pearson correlation coefficient, mean absolute error (MAE), and one-dimensional Wasserstein distance. The complete synthetic-signal generation and quantitative evaluation procedure was independently repeated five times using different diffusion-noise realizations. Unless otherwise specified, the correlation, MAE, and Wasserstein-distance results are reported as mean ± SD across the five generation runs. Accordingly, the same-condition analysis was used specifically to test for deterministic or near-duplicate generation under fixed conditions; it was not interpreted as evidence that the full real conditional distribution had been recovered.

#### 2.8.7. Synthetic-to-Real Generalization Analysis

To evaluate whether the CDM-generated PPG signals contained information that could transfer to real unseen participants, an additional synthetic-to-real generalization analysis was performed using the original seed-42 subject-wise five-fold partition. The same regression architecture and training settings were used under three training-data configurations: real PPG only, synthetic PPG only, and real plus synthetic PPG. In each outer fold, synthetic training signals were generated exclusively from the physiological conditions of the outer-training participants using the corresponding fold-specific CDM. The outer-test fold contained only real PPG recordings and remained inaccessible during diffusion-model training, synthetic-data generation, and regression-model training. In the synthetic-only configuration, the regression model was trained without real PPG waveforms; in the combined configuration, real and synthetic PPG signals were used together according to the augmentation protocol described above. For all three configurations, the two predictions from the non-overlapping 5 s segments of each original 10 s recording were averaged to obtain one recording-level prediction. The five outer-test folds were then pooled to yield 67 recording-level out-of-fold predictions, from which RMSE, MAE, MARD, and Clarke Zone A and A + B proportions were calculated. This experiment was intended to assess the transferability of synthetic PPG features to real held-out participants rather than to evaluate synthetic data as a replacement for real physiological measurements. The synthetic-only configuration therefore served as a cross-domain transfer test: if label-specific patterns were useful only within the synthetic domain, performance would be expected to deteriorate substantially when evaluated on real outer-test recordings. Conversely, any retained performance was interpreted cautiously as evidence that at least part of the generated predictive structure transferred to real PPG.

#### 2.8.8. Participant-Clustered Paired Bootstrap Confidence-Interval Analysis

To quantify the uncertainty of the direct comparison between the original CBHFF and improved CMAB, participant-clustered paired bootstrap resampling was applied to their matched out-of-fold predictions. The two 5 s predictions originating from each original 10 s recording were first averaged to obtain recording-level predictions. Participants, rather than individual recordings, were then resampled with replacement so that all recordings belonging to a sampled participant were retained together. The same resampled participant set was used simultaneously for both models, thereby preserving the paired structure of the comparison. For each resample, the difference was defined as Δ = metric_CMAB − metric_CBHFF. The 95% confidence interval was obtained from the 2.5th and 97.5th percentiles of the empirical distribution of the paired differences. Negative differences favor CMAB for RMSE, MAE, and MARD, whereas positive differences favor CMAB for Clarke Zone A. Because all model comparisons used the same outer-fold participant assignments, each CBHFF prediction was paired with the CMAB prediction for the same original recording. Confidence intervals excluding zero were interpreted as evidence of a paired difference, whereas intervals including zero were not interpreted as statistically supported differences.

## 3. Results

### 3.1. Effect of Data Augmentation Strategies

To determine whether conditional diffusion augmentation provided greater benefit than conventional signal transformations, the same baseline BiLSTM model was trained using the three data configurations described in Section 2.5.5. Because this experiment compares augmentation strategies at the 5 s model-input level under identical subject-wise splits, Table 3 is reported as a secondary segment-level analysis.

In this secondary segment-level comparison, conventional augmentation reduced the mean RMSE from 0.87 to 0.82 mmol/L, the mean MAE from 0.67 to 0.64 mmol/L, and the mean MARD from 13.71% to 12.44%, while increasing the mean Clarke Zone A proportion from 86.4% to 88.1% and the mean Zone A + B proportion from 98.5% to 99.0%. CDM-based augmentation produced the largest numerical improvement among the three evaluated training-data configurations, reducing the mean RMSE to 0.73 mmol/L, the mean MAE to 0.58 mmol/L, and the mean MARD to 8.05%, while increasing the mean Clarke Zone A proportion to 90.8% and Zone A + B to 100.0%. These results compare augmentation strategies under a common segment-level evaluation unit and matched augmentation volume; they are not treated as independent glucose-measurement estimates or as proof that one augmentation strategy is universally superior.

### 3.2. Cross-Validation Performance

Table 4 reports the fold-wise segment-level performance of the proposed full model under subject-wise five-fold cross-validation and, in its final row, the primary pooled recording-level out-of-fold performance. The fold-specific segment-level values are retained as a secondary analysis, whereas the pooled recording-level row summarizes the 67 original 10 s recording/reference-measurement pairs from 23 participants.

Across the five outer test folds, the secondary segment-level RMSE ranged from 0.65 to 0.70 mmol/L, MAE ranged from 0.48 to 0.53 mmol/L, and MARD ranged from 7.31% to 7.86%. The corresponding fold-wise mean values were 0.68 ± 0.02 mmol/L for RMSE, 0.50 ± 0.02 mmol/L for MAE, and 7.58 ± 0.22% for MARD. The mean segment-level Clarke Zone A proportion was 94.8 ± 2.11%, and all five folds had 100.0% of segment-level predictions in Zones A + B. Because these five fold-level summaries are secondary observations from a single participant-wise partition, they are not used as evidence of statistical stability.

For the primary analysis, the two out-of-fold predictions corresponding to each original 10 s recording were averaged, yielding 67 recording-level predictions matched to 67 recording-level reference glucose measurements from 23 participants. The proposed model achieved an RMSE of 0.59 mmol/L, an MAE of 0.38 mmol/L, and a MARD of 5.48%. In the Clarke error-grid analysis, 65 of 67 predictions (97.0%) were located in Zone A, and all 67 predictions (100%) were located in Zones A + B. These values constitute the primary seed-42 recording-level performance results. The complete evaluation was repeated with participant-level fold assignments generated using seeds 2026 and 3407 only as a partition-sensitivity analysis, as reported below. Because some participants contributed multiple recordings, the 67 recording-level observations are not interpreted as 67 independent participant-level samples; participant-level dependence is addressed through participant-clustered resampling for inferential comparisons. These pooled out-of-fold metrics were calculated directly from the 67 recording-level predictions across all five held-out folds rather than from an unweighted average of the five fold-wise summary values.

To examine whether the final performance depended strongly on the participant assignment generated with seed 42, the complete full-model evaluation was repeated using two additional participant-level fold assignments. For seeds 42, 2026, and 3407, respectively, the recording-level results were as follows: RMSE, 0.59, 0.60, and 0.61 mmol/L; MAE, 0.38, 0.39, and 0.39 mmol/L; MARD, 5.48%, 5.52%, and 5.55%; Clarke Zone A, 97.0%, 96.8%, and 96.6%; and Clarke Zones A + B, 100%, 100%, and 100%. Across the three participant-level fold assignments, the corresponding mean ± sample SD values were 0.60 ± 0.01 mmol/L for RMSE, 0.39 ± 0.01 mmol/L for MAE, 5.52 ± 0.04% for MARD, 96.8 ± 0.2% for Clarke Zone A, and 100 ± 0.0% for Clarke Zones A + B. These descriptive results were similar across the three participant-level fold assignments, indicating that the observed performance was not confined to the original seed-42 partition. However, the three-partition summary is not interpreted as a formal uncertainty interval, and uncertainty remains because of the limited cohort size.

### 3.3. Stepwise Ablation and Package-Level CMAB Comparison

To evaluate the relative effects of the principal framework components under a common model-input unit, we performed a stepwise ablation study at the 5 s segment level. The baseline model consists of a basic BiLSTM network using only the original data and does not incorporate CDM, the CMAB architecture, or MFO optimization. Building on this baseline, CDM, the complete CMAB architecture, and MFO-based hyperparameter optimization were introduced sequentially. Because all variants use identical subject-wise partitions and the same segment-level evaluation unit, Table 5 is presented as a secondary within-study comparison rather than as the primary estimate of glucose-prediction performance. Importantly, the present experiments do not remove the internal CMAB modifications one at a time; therefore, they evaluate CMAB only as a package and cannot attribute performance changes to channel attention, temporal attention, the cascaded BiLSTM backbone, multiplicative attention fusion, or cross-layer interaction individually.

As shown in Table 5, the secondary segment-level comparison showed that introducing CDM into the baseline BiLSTM reduced the mean RMSE from 0.87 ± 0.04 to 0.73 ± 0.03 mmol/L and the mean MARD from 13.71 ± 0.65% to 8.05 ± 0.31%, while the mean Clarke Zone A proportion increased from 86.4 ± 1.2% to 90.8 ± 1.91%.

The original CBHFF and improved CMAB were further compared under the same CDM-augmented training setting without MFO. The improved CMAB showed modest numerical reductions in MAE (0.54 ± 0.03 to 0.51 ± 0.02 mmol/L) and MARD (7.85 ± 0.31% to 7.69 ± 0.28%), while the mean Clarke Zone A proportion increased from 91.6 ± 2.32% to 92.9 ± 1.86%; the mean RMSE remained 0.71 ± 0.03 mmol/L. Because several architectural modifications were introduced together, this comparison reflects their collective package-level effect and does not isolate the contribution of any single CMAB component. In particular, no isolated superiority is claimed for the local cross-channel attention operator, temporal self-attention branch, multiplicative attention fusion, cascaded BiLSTM backbone, or cross-layer interaction mechanism. The identical rounded segment-level fold-mean RMSE values in Table 5 do not imply identical out-of-fold predictions. Accordingly, inferential comparison between CBHFF and CMAB was based on the matched recording-level out-of-fold predictions rather than on the five fold means.

To assess whether these modest numerical differences were supported by paired uncertainty estimates, a participant-clustered paired bootstrap analysis was performed on the matched recording-level out-of-fold predictions. The paired RMSE difference was −0.02 mmol/L (95% CI: −0.05 to 0.02), and the Clarke Zone A difference was +1.2 percentage points (95% CI: −1.5 to 3.8); both confidence intervals included zero. In contrast, CMAB showed a reduction in MAE of −0.02 mmol/L (95% CI: −0.04 to −0.01) and in MARD of −0.30 percentage points (95% CI: −0.55 to −0.06), with both confidence intervals excluding zero. Thus, the paired analysis supports reductions in MAE and MARD, while the RMSE and Zone A differences should be interpreted as numerical rather than statistically supported improvements.The participant-clustered paired bootstrap results are summarized in Table 6.

**Table 6:** Participant-clustered paired bootstrap comparison between the original CBHFF and improved CMAB.

Metric	Paired Difference (CMAB − CBHFF)	95% CI
RMSE (mmol/L)	−0.02	−0.05 to 0.02
MAE (mmol/L)	−0.02	−0.04 to −0.01
MARD (percentage points)	−0.30	−0.55 to −0.06
Clarke Zone A (percentage points)	+1.2	−1.5 to 3.8

Note: Negative differences in RMSE, MAE, and MARD favor CMAB, whereas positive differences in Clarke Zone A favor CMAB. Confidence intervals were obtained using participant-clustered paired bootstrap resampling of the matched recording-level out-of-fold predictions. The paired analysis used the same 67 recording-level out-of-fold observations for both models; participant clustering preserved within-participant dependence during resampling.

Finally, the secondary segment-level results after MFO-based hyperparameter optimization were an RMSE of 0.68 ± 0.02 mmol/L, a MARD of 7.58 ± 0.22%, and a Clarke Zone A proportion of 94.8 ± 2.11%. The primary recording-level performance of the full model is reported in Section 3.2.

### 3.4. Contextual Summary of Previously Reported PPG-Based Methods

To provide descriptive context only, Table 7 summarizes representative PPG-based blood glucose estimation studies together with the present recording-level results. The revised table now makes the major sources of cross-study heterogeneity explicit by reporting cohort/data scale, signal modality and sensing context, glucose range and validation design, as well as the type of error grid used. These studies differ substantially in participant/sample size, sensor wavelength and hardware, input modality, glucose distribution, validation protocol, and statistical unit of evaluation; therefore, the reported numerical values are not treated as a controlled head-to-head ranking.

**Figure 10:**
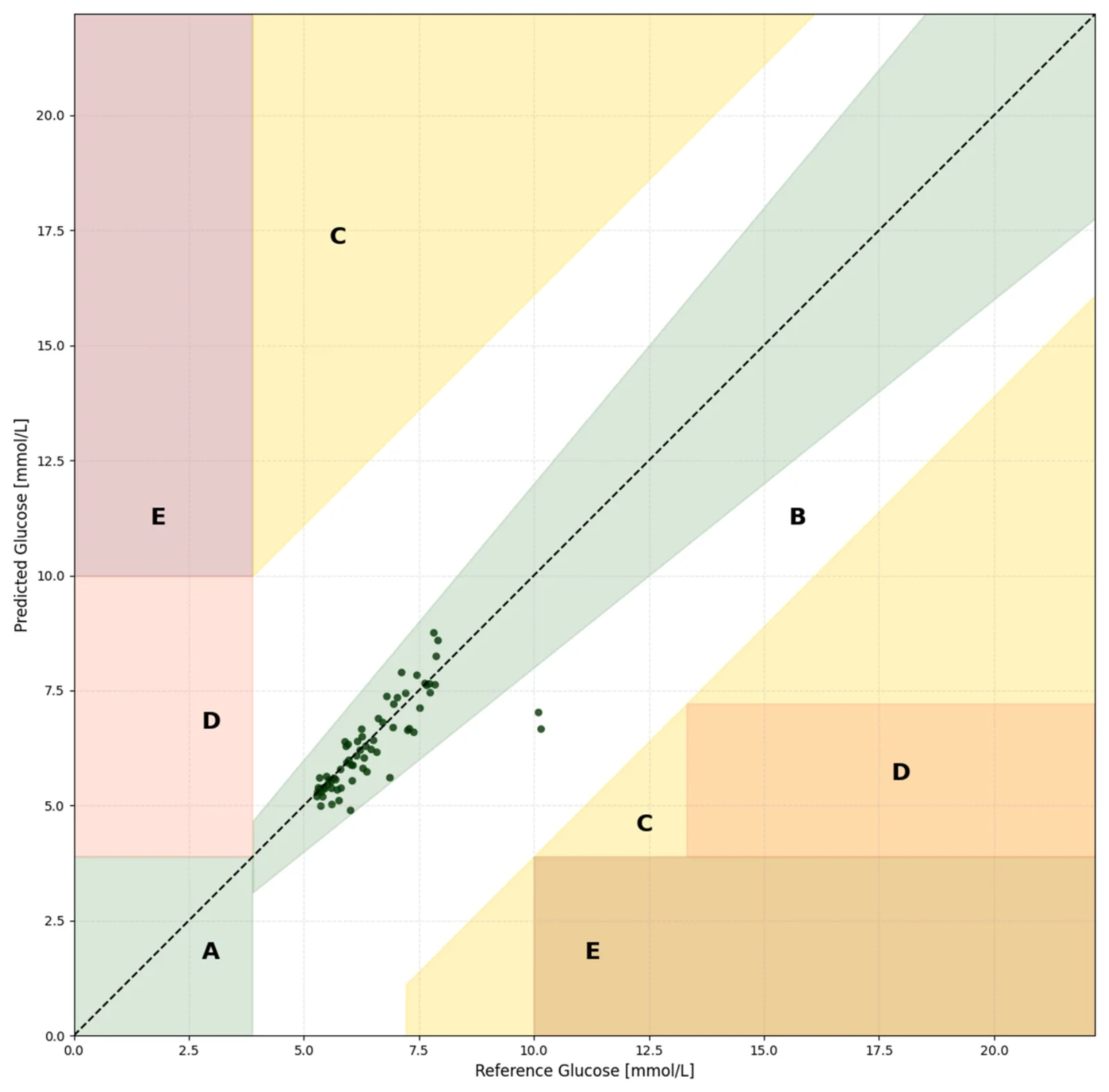
Clarke error-grid analysis for the primary seed-42 participant partition (n = 67 recording-level out-of-fold predictions from 23 participants; each point corresponds to one original 10 s recording/reference measurement; reference and predicted glucose are shown in mmol/L). The black dashed line denotes perfect agreement (y = x). The letters A–E denote the five Clarke error-grid zones, and the different background colors are used to visually distinguish these zones.

Figure 10 shows the Clarke error-grid analysis for the primary seed-42 participant partition based on 67 recording-level out-of-fold predictions. Each point represents one original 10 s PPG recording and its corresponding recording-level reference glucose measurement, not an individual 5 s segment. The recording-level prediction was obtained by averaging the predictions from the two corresponding 5 s segments. Some participants contribute multiple points; therefore, these recording-level observations are not assumed to be mutually independent across participants, and participant-clustered resampling is used for inferential uncertainty analyses. Of the 67 recording-level predictions, 65 (97.0%) fell within Zone A, and all 67 (100%) fell within Zones A + B. Because the observed reference-glucose range was 4.89–10.17 mmol/L, this error-grid result does not evaluate performance in hypoglycemia and provides no evidence of safety in marked hyperglycemia.

### 3.5. Morphological Fidelity of Generated PPG Signals

In addition to evaluating the effect of synthetic data on blood glucose prediction, the local waveform morphology of representative real and generated PPG segments was examined. The analysis focused on the organization of local extrema, the spacing between detected peaks, and the temporal characteristics of the rising and falling phases.

**Figure 11:**
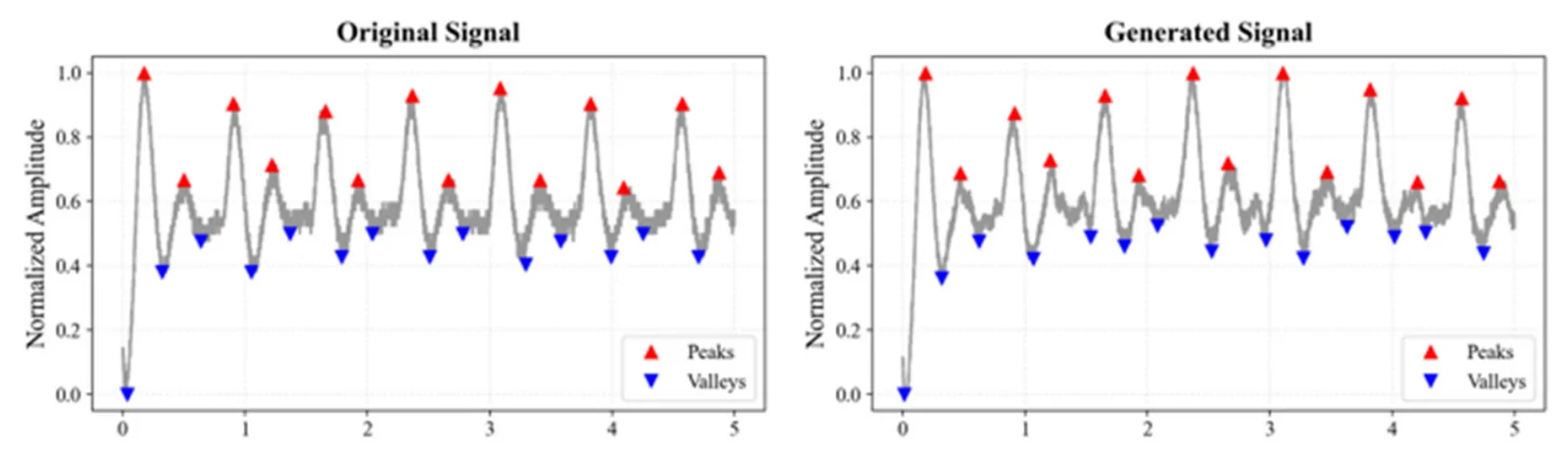
Comparison of detected local peaks and valleys in representative real and generated PPG segments. Peaks were identified with scipy.signal.find_peaks using height = max(0.1, 0.5 × SD(signal)) and a minimum distance of 543 samples (0.25 s); valleys were the local minima between adjacent peaks, with the leading pre-peak minimum retained as the initial valley.

As shown in Figure 11, both signals exhibit an alternating sequence of dominant and secondary peaks together with intervening valleys, indicating that the CDM preserves the basic local organization of the PPG waveform. The approximate number and timing of detected extrema are broadly comparable, although the generated signal exhibits greater short-term fluctuation and small differences in peak amplitude. Because the detector identifies both dominant systolic peaks and smaller secondary peaks, these markers are used to characterize local waveform morphology rather than to count cardiac beats.

**Figure 12:**
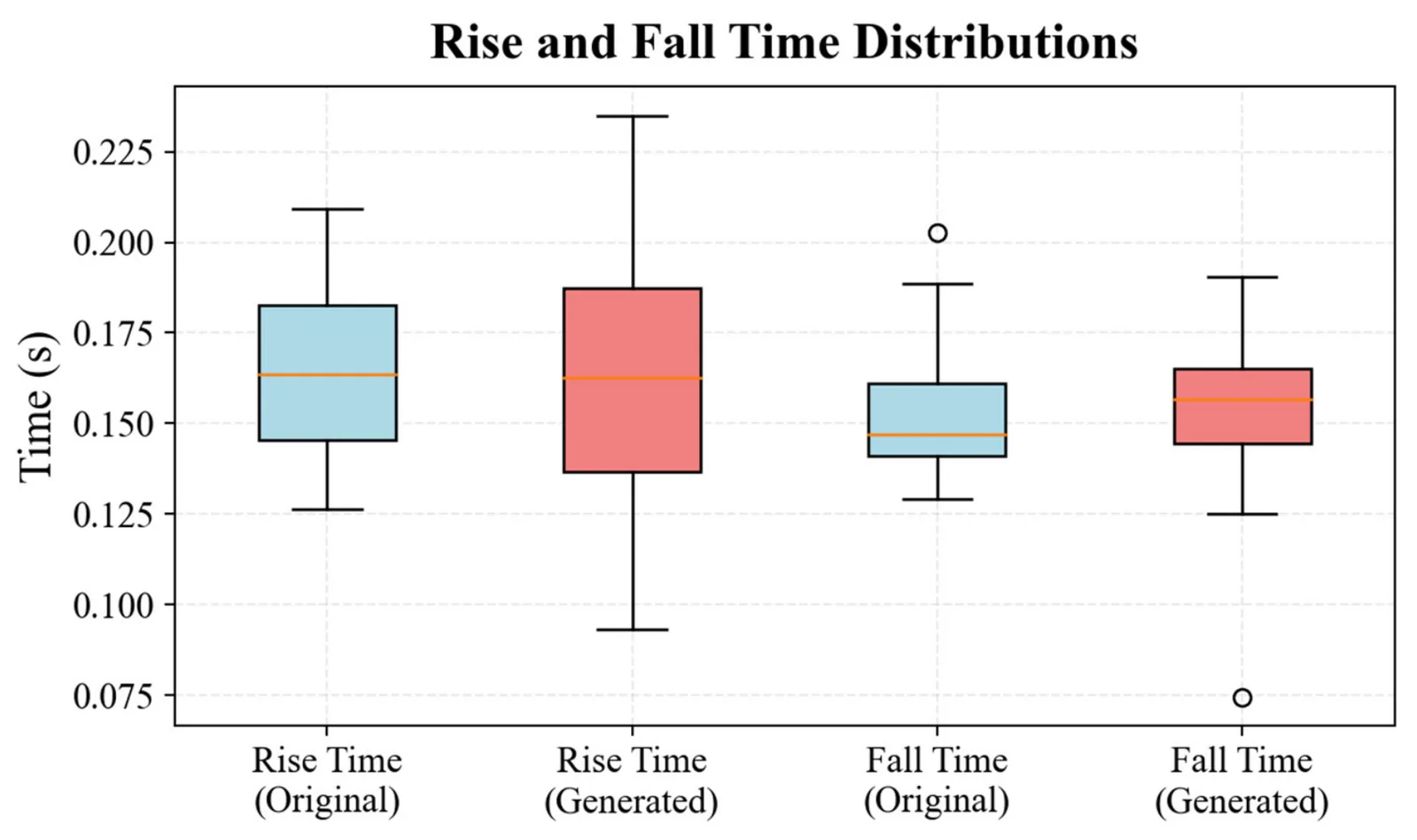
Comparison of rise-time and fall-time distributions between real and conditionally generated PPG signals. Boxplot sample sizes are n = 14, 14, 13, and 13 for real rise time, generated rise time, real fall time, and generated fall time, respectively. These observations are detected within the representative 5 s waveforms in Figure 11 and are descriptive interval-level measurements rather than independent participants or glucose measurements.

As shown in Figure 12, the median rise times of the real and generated signals are similar, whereas the generated group presents a somewhat wider distribution. The median fall times are also close, although a low-duration outlier is observed in the generated group. Overall, the generated signals preserved the main local pulse morphology while introducing waveform diversity.

### 3.6. Heart-Rate Conditional Consistency of Generated PPG Signals

**Figure 13:**
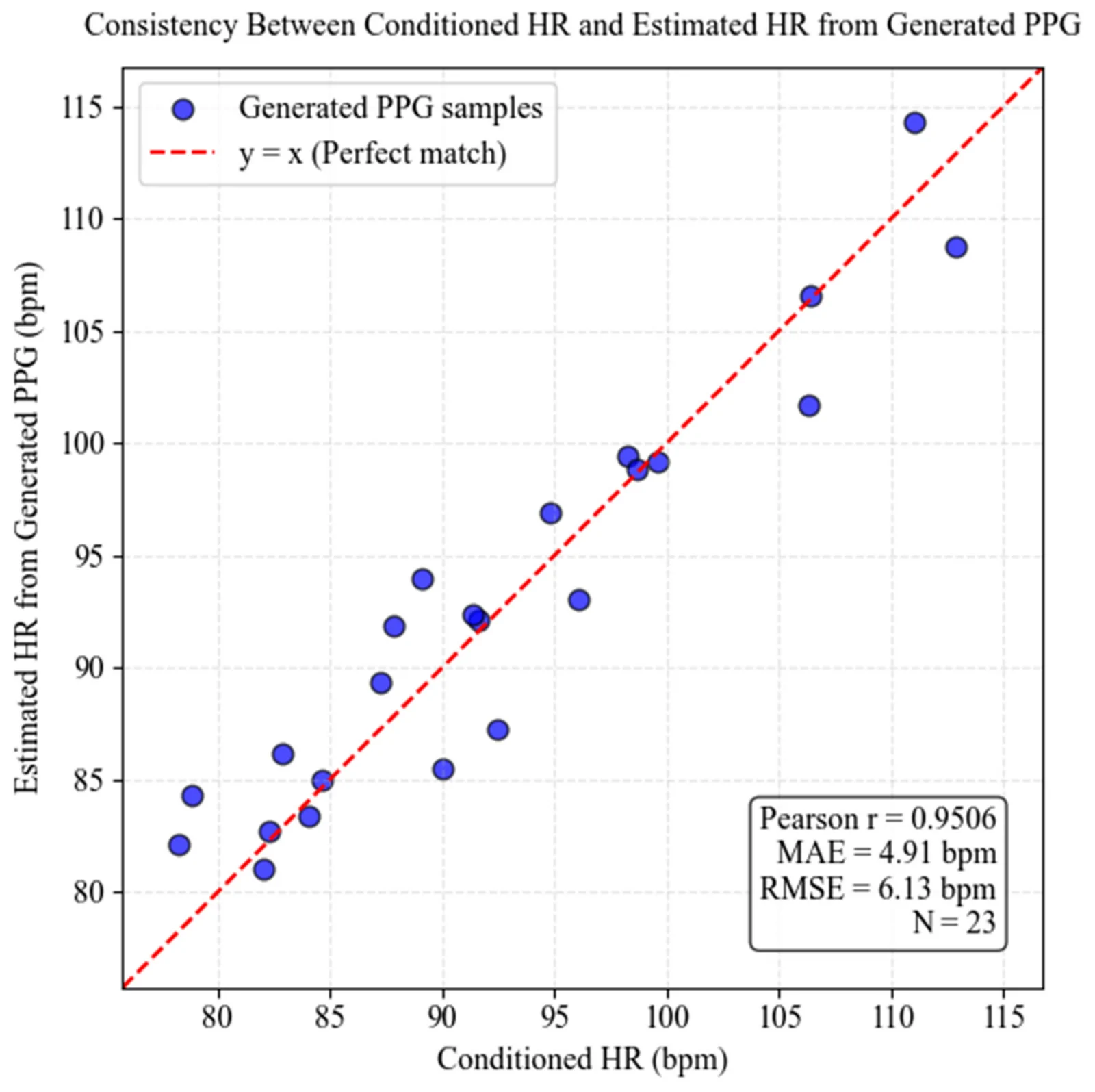
Consistency between conditioned heart rate (HR) and HR estimated from conditionally generated PPG signals. Each point represents one generated PPG sample (N = 23). The red dashed line denotes perfect agreement (y = x).

To quantitatively assess whether the CDM preserved the specified HR condition, the pulse rate estimated from the generated PPG signals was compared with the HR supplied to the diffusion model. As shown in Figure 13, the estimated HR exhibited a strong correlation with the conditioning HR across 23 generated samples (Pearson’s r = 0.9506). The corresponding MAE and RMSE were 4.91 bpm and 6.13 bpm, respectively. These results indicate that the generated waveforms retained substantial HR-related temporal information. The remaining deviations from the identity line also show that the target HR was not reproduced perfectly; therefore, this analysis is interpreted as evidence of HR-conditional consistency rather than complete physiological equivalence.

### 3.7. BGL Conditional Responsiveness of Generated PPG Signals

**Figure 14:**
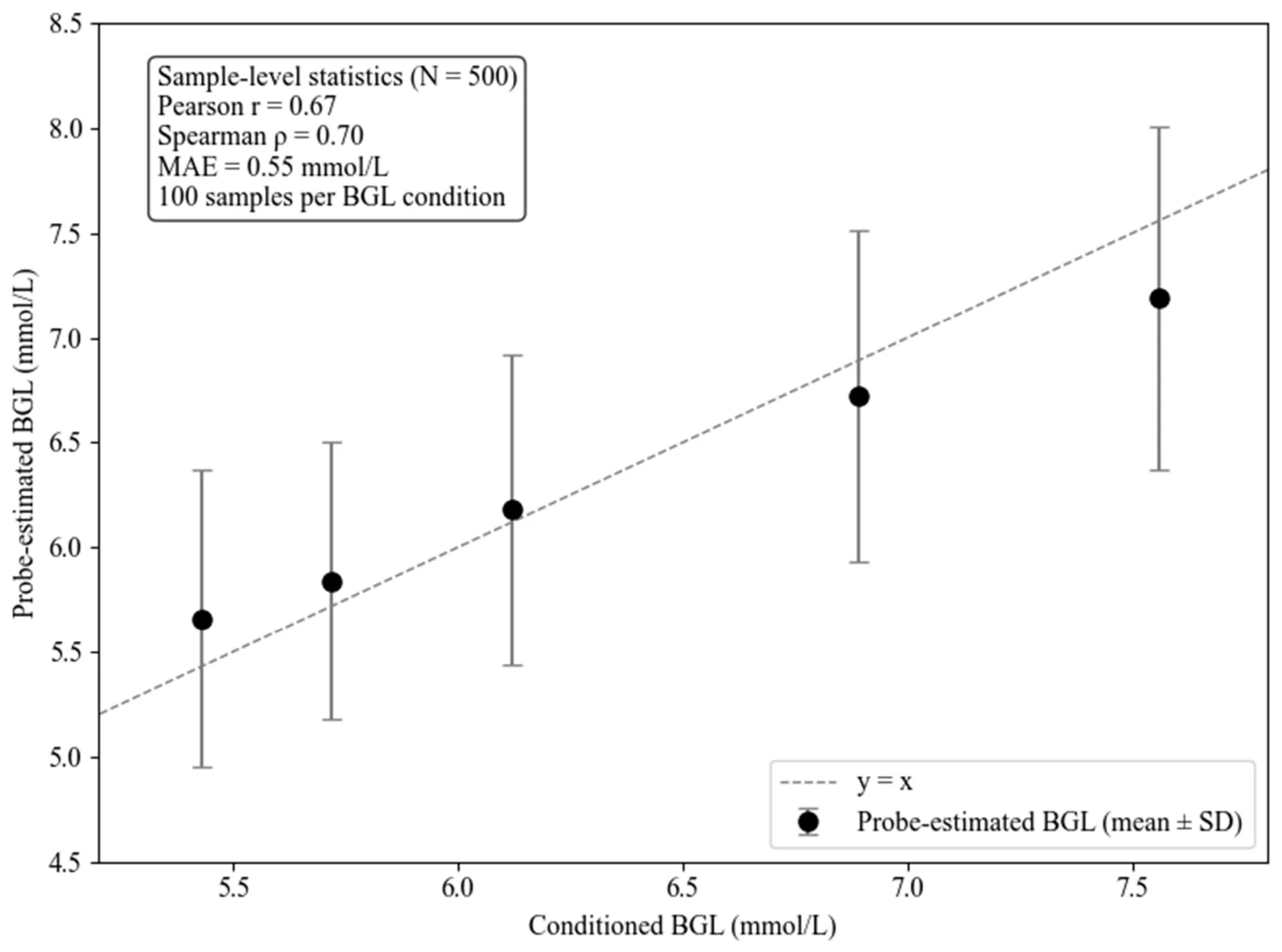
BGL conditional responsiveness of the generated PPG signals. HR and BMI were fixed at 91.38 bpm and 21.45 kg/m^2^, respectively, while BGL was varied across five representative levels. Each point and error bar represents the mean ± SD of probe-estimated BGL for 100 generated PPG samples at the corresponding condition. The dashed line denotes perfect agreement (y = x).

To further assess whether the CDM responded systematically to the BGL condition, HR and BMI were held constant while BGL was varied across five representative levels. As shown in Figure 14, the mean probe-estimated BGL increased progressively from 5.66 ± 0.71 to 7.19 ± 0.82 mmol/L as the conditioning BGL increased from 5.43 to 7.56 mmol/L. The mean probe-estimated BGL increased progressively from 5.66 ± 0.71 to 7.19 ± 0.82 mmol/L as the conditioning BGL increased from 5.43 to 7.56 mmol/L. Specifically, the probe-estimated values were 5.66 ± 0.71, 5.84 ± 0.66, 6.18 ± 0.74, 6.72 ± 0.79, and 7.19 ± 0.82 mmol/L for conditioning BGL values of 5.43, 5.72, 6.12, 6.89, and 7.56 mmol/L, respectively. Across all 500 generated samples, the conditioned and probe-estimated BGL values showed a moderate positive association (Pearson’s r = 0.67; Spearman’s ρ = 0.70), with an MAE of 0.55 mmol/L. These findings provide quantitative evidence that changing the BGL condition while holding HR and BMI constant produced systematic changes in the generated PPG signals that were recognizable by a model trained only on real PPG data. Because the probe had been trained exclusively on real PPG and had never seen CDM-generated signals, the monotonic response suggests that the BGL-conditioned waveform changes overlapped with features learned from real data. It does not, however, exclude all generator-specific label artifacts or establish a causal optical glucose signature.

### 3.8. Same-Condition Diversity and Distribution-Level Consistency

Across the repeated generation experiments, PPG realizations generated under identical physiological conditions remained highly similar while retaining non-zero stochastic variation. The mean pairwise Pearson correlation was 0.94 ± 0.04, and the normalized Euclidean distance was 0.35 ± 0.06. These results indicate that the CDM produced condition-consistent but non-identical waveform realizations rather than deterministic copies.

As summarized in Table 8, the generated signals closely reproduced the global periodic characteristics of the real PPG signals. Dominant frequency was 1.59 ± 0.17 Hz for both real and generated PPG, with a Pearson correlation of 0.98 ± 0.01 and an MAE of 0.015 ± 0.008 Hz. Mean peak interval was similarly preserved (653 ± 61 ms versus 654 ± 62 ms; r = 0.97 ± 0.02; MAE = 5 ± 3 ms). Agreement remained strong for local waveform morphology, although the correlations were slightly lower for rise time (r = 0.91 ± 0.03) and fall time (r = 0.90 ± 0.03). The Wasserstein distances were small for all four features and increased from global periodic features to local rise/fall characteristics. Collectively, these findings indicate close distribution-level agreement for the principal PPG temporal features while allowing greater variability in local waveform morphology.

### 3.9. Synthetic-to-Real Generalization on Real Held-Out Participants

The synthetic-to-real generalization analysis compared models trained with real PPG only, synthetic PPG only, and the combination of real and synthetic PPG, while all evaluations were performed on the same real held-out participants. The resulting recording-level performance is summarized in Table 9.

Training on real PPG alone achieved an RMSE of 0.63 mmol/L, an MAE of 0.41 mmol/L, and a MARD of 5.89%, with 64 of 67 predictions (95.5%) in Clarke Zone A and 66 of 67 (98.5%) in Zones A + B. When the regression model was trained exclusively on synthetic PPG generated from the physiological conditions of the outer-training participants, performance decreased to an RMSE of 1.14 mmol/L, an MAE of 0.88 mmol/L, and a MARD of 12.70%; 51 of 67 predictions (76.1%) were in Zone A and 63 of 67 (94.0%) were in Zones A + B. Despite this reduction, the synthetic-only model retained measurable predictive capability on entirely real held-out participants. Combining real and synthetic training data produced the best performance, with an RMSE of 0.59 mmol/L, an MAE of 0.38 mmol/L, a MARD of 5.48%, 65 of 67 predictions (97.0%) in Zone A, and all 67 predictions (100%) in Zones A + B. These results indicate that the generated PPG signals are more appropriately interpreted as complementary augmentation data that enrich training variability rather than as substitutes for independent real physiological measurements. The marked performance gap between synthetic-only and real-only training also argues against interpreting the generator as having created a synthetic shortcut sufficient for high-accuracy prediction on real data. At the same time, the non-zero transfer performance and the further improvement obtained by combining real and synthetic data suggest partial overlap between the generated and real predictive structure.

### 3.10. Matched-Budget Comparison of MFO and Random Search

A matched-budget Random Search experiment was conducted to determine whether MFO provided numerical benefits beyond generic hyperparameter tuning. For each outer fold, Random Search used exactly the same hyperparameter search space and the same number of candidate evaluations actually consumed by MFO after early stopping (160, 190, 200, 150, and 180 evaluations for Folds 1–5, respectively). The best inner-validation RMSE values obtained by Random Search were 0.69, 0.68, 0.70, 0.66, and 0.71 mmol/L, and the corresponding outer-test RMSE values were 0.65, 0.63, 0.66, 0.62, and 0.68 mmol/L.The recording-level comparison between MFO and Random Search is summarized in Table 10.

**Table 10:** Recording-level comparison of MFO and Random Search under matched hyperparameter-evaluation budgets.

Optimization	RMSE (mmol/L)	MAE (mmol/L)	MARD (%)	Zone A (%)	Zones A + B (%)
Random Search	0.63	0.41	5.89	94.0 (63/67)	98.5 (66/67)
MFO	**0.59**	**0.38**	**5.48**	**97.0 (65/67)**	**100 (67/67)**

Note: Metrics were calculated from pooled recording-level out-of-fold predictions for the primary seed-42 participant partition. Random Search and MFO used identical inner participant partitions, hyperparameter ranges, model-training procedures, and fitness criteria. The Random Search budget was matched to the actual MFO candidate-evaluation count in each outer fold. Values in parentheses indicate the number of recordings out of 67.

At the pooled recording level, Random Search achieved an RMSE of 0.63 mmol/L, an MAE of 0.41 mmol/L, and a MARD of 5.89%, with 63 of 67 predictions (94.0%) in Clarke Zone A and 66 of 67 (98.5%) in Zones A + B. Under the matched search budget, MFO achieved an RMSE of 0.59 mmol/L, an MAE of 0.38 mmol/L, and a MARD of 5.48%, with 65 of 67 predictions (97.0%) in Zone A and all 67 predictions (100%) in Zones A + B. Thus, MFO yielded consistently better numerical results across the evaluated recording-level metrics in this matched-budget comparison. These findings support the choice of MFO for the present experimental setting, but they are treated as a within-study numerical benchmark rather than as an optimizer-level significance test or evidence of universal superiority over alternative tuning strategies.

## 4. Discussion

The proposed framework integrates conditional diffusion augmentation, a cascaded multi-view attention BiLSTM network, and MFO-based hyperparameter optimization for PPG-based blood glucose estimation. In the primary seed-42 recording-level analysis, the model achieved an RMSE of 0.59 mmol/L, an MAE of 0.38 mmol/L, a MARD of 5.48%, and a Clarke Zone A proportion of 97.0%, with 100% in Zones A + B. To assess sensitivity to participant-fold assignment, the complete evaluation was repeated using seeds 2026 and 3407; across the three participant-level assignments, the descriptive mean ± sample SD was 0.60 ± 0.01 mmol/L for RMSE, 0.39 ± 0.01 mmol/L for MAE, 5.52 ± 0.04% for MARD, 96.8 ± 0.2% for Clarke Zone A, and 100 ± 0.0% for Zones A + B. The similar results obtained with the two additional participant-level fold assignments reduce concern that the primary finding was specific to the seed-42 partition, although the small cohort still limits the precision of uncertainty estimates. The fold-wise and ablation results in Table 3, Table 4 and Table 5 are retained as secondary segment-level analyses for within-study comparison and are not interpreted as independent glucose-measurement estimates. The cross-study literature summary in Table 7 is used only to provide descriptive context. Because the cited studies were conducted with different datasets, sensors/wavelengths, glucose ranges, sample sizes, splitting strategies, and validation protocols, their reported metrics are not used to establish a direct performance ranking or a state-of-the-art superiority claim for the present method.

Clinical interpretation is necessarily limited by the study population and data source. The public dataset contains only 23 participants measured with a single acquisition system, and the recording-level reference BGL values span 4.89–10.17 mmol/L. Thus, no reference measurements fall in the hypoglycemic range (<3.9 mmol/L), and the data do not cover marked hyperglycemia. In addition, the model was not evaluated on an independent external dataset. Consequently, the observed Clarke Zone A and Zone A + B proportions describe agreement only within this restricted study distribution and cannot establish clinical utility, treatment safety, or reliability across the full clinically relevant glucose range. External validation on larger, clinically characterized cohorts spanning hypoglycemia, normoglycemia, and substantial hyperglycemia, ideally across independent devices and acquisition sites, is required before deployment-oriented or clinical claims can be made.

The statistical unit of the primary performance analysis is the original 10 s recording/reference-measurement pair rather than the derived 5 s segment. Because the 67 recording-level measurements were contributed by only 23 participants, repeated recordings within a participant may remain correlated and are not treated as independent participant-level samples. A single glucose value was not formed by averaging all recordings within each participant because the public dataset provides recording-specific reference labels rather than one participant-level target. Where inferential uncertainty is required, participant-clustered resampling retains all recordings from a sampled participant together so that this within-participant dependence is preserved.

Recent 2026 studies provide useful methodological context for these findings. Soliman et al. used cross-domain transfer learning from a large PPG–blood pressure dataset to the small Mazandaran PPG–BGL dataset, demonstrating an alternative strategy for addressing limited glucose-labeled PPG data through transferable hemodynamic representations [17]. The present work considers the same limited-data setting from a complementary direction by using condition-guided diffusion augmentation rather than cross-domain pretraining, with the aim of increasing waveform variation around observed training labels rather than increasing the number of independent glucose measurements. Msokar et al., although focused on blood-pressure staging, showed that interpretable multi-domain PPG features, training-only preprocessing transformations, and explicit attention to validation design are important when deriving physiological predictions from small or imbalanced PPG datasets [18]. Together, these recent studies reinforce the need to combine data-efficiency strategies with leakage control, physiological plausibility, and cautious interpretation. Because their prediction targets, datasets, and validation protocols differ from those used here, they are discussed as methodological context rather than direct performance comparators.

The secondary segment-level ablation results suggest that CDM provided the largest numerical reduction in RMSE among the sequential additions examined in this study. By incorporating BGL, HR, and BMI into the denoising process, the diffusion model generated additional PPG waveform realizations associated with physiological states already represented in the training data. The generated signals retained the main peak–valley organization and exhibited similar rise- and fall-time characteristics to the real signals. These findings support the use of condition-guided augmentation to increase waveform diversity while not implying that synthetic generation creates new independent glucose measurements.

Quantitative HR-condition validation further showed a strong association between the HR supplied to the CDM and the pulse rate estimated from generated PPG signals (Pearson’s r = 0.9506; MAE = 4.91 bpm; RMSE = 6.13 bpm). This result provides additional evidence that the generator responds to the HR condition. However, the non-zero errors indicate that the target HR is not reproduced exactly, and this analysis alone should not be interpreted as proof of complete physiological fidelity across all conditioning variables.

The BGL-condition analysis provided complementary evidence of conditional responsiveness. With HR and BMI held fixed, the probe-estimated BGL increased monotonically across the five conditioning levels, with Pearson’s r = 0.67, Spearman’s ρ = 0.70, and an MAE of 0.55 mmol/L across the 500 generated samples. The association was weaker than that observed for HR conditioning, and the probe estimates showed some compression toward the central BGL range, particularly at the highest conditioning level. Therefore, these results support systematic BGL-related responsiveness of the generated waveforms but should not be interpreted as proof that the CDM reproduces the complete physiological relationship between glucose concentration and PPG morphology.

Importantly, the predictive relationship examined here should be interpreted as a statistical and physiological association rather than evidence of a direct optical glucose-sensing mechanism. The green 550 nm PPG signal in this dataset primarily reflects pulsatile blood-volume and vascular optical changes, and any BGL-related patterns available to the model may arise indirectly through coupled hemodynamic or metabolic variation. Such patterns can also be affected by heart rate, blood pressure, tissue perfusion, skin temperature, sensor–skin contact, and skin-dependent optical properties. The present experiments therefore do not identify a glucose-specific optical signature or establish a causal mechanism linking glucose concentration to the observed PPG waveform.

The additional diversity and distribution-level analyses further support this interpretation. Under identical physiological conditions, the generated PPG signals showed high pairwise similarity (r = 0.94 ± 0.04) together with a non-zero normalized Euclidean distance (0.35 ± 0.06), indicating preservation of condition-specific structure without exact waveform duplication. Across five independent generation runs, dominant frequency and peak interval showed the strongest real–generated agreement, whereas rise- and fall-time correlations were slightly lower. This pattern suggests that the CDM preserves global periodic organization more strongly than fine local morphology. Accordingly, the generated data should be interpreted as condition-consistent waveform augmentation rather than physiologically exact replicas of real PPG signals.

The synthetic-to-real experiment provided an additional test of whether the generated waveforms contained information transferable to real unseen participants. A model trained only on synthetic PPG performed worse than the corresponding real-only model (RMSE 1.14 versus 0.63 mmol/L; MARD 12.70% versus 5.89%), indicating that synthetic data cannot replace real physiological measurements. Nevertheless, the synthetic-only model retained non-zero predictive capability on completely real held-out participants, and combining real and synthetic training data improved the recording-level RMSE from 0.63 to 0.59 mmol/L and the MARD from 5.89% to 5.48%. Together with the conditional and distribution-level analyses, these findings support the interpretation of CDM-generated PPG as a source of complementary waveform variation for augmentation and regularization while also highlighting the remaining gap between generated and real PPG distributions. Taken together, the fixed-HR/BMI BGL perturbation, same-condition diversity analysis, distribution-level feature comparison, and synthetic-to-real transfer test address the concern about artificial label-specific patterns from complementary perspectives. Nevertheless, none of these analyses proves that the CDM recovered the complete physiological conditional distribution p(PPG|BGL, HR, BMI), and residual generator-specific artifacts cannot be excluded.

Two CDM design choices should be interpreted cautiously. First, the real-to-synthetic ratio was fixed at 1:2, and no dedicated ratio-sensitivity experiment was performed; therefore, the present results do not establish that 1:2 is optimal or that the augmentation gain is invariant with respect to the amount of synthetic data. Second, λ = 0.3 was used as a fixed SSIM regularization weight to keep the diffusion MSE term dominant, but the study did not isolate the SSIM term in a dedicated loss ablation. Accordingly, the observed benefit of CDM cannot be attributed specifically to SSIM, and both augmentation-ratio sensitivity and loss-component ablation remain important topics for future validation.

The CMAB comparison showed modest numerical improvements over the capacity-matched CBHFF configuration under the same secondary segment-level evaluation setting. The participant-clustered paired analysis of the matched recording-level out-of-fold predictions supported reductions in MAE (Δ = −0.02 mmol/L, 95% CI: −0.04 to −0.01 mmol/L) and MARD (Δ = −0.30 percentage points, 95% CI: −0.55 to −0.06), whereas the confidence intervals for RMSE (Δ = −0.02 mmol/L, 95% CI: −0.05 to 0.02 mmol/L) and Clarke Zone A (Δ = +1.2 percentage points, 95% CI: −1.5 to 3.8) included zero. Accordingly, the evidence does not support a uniform improvement across all metrics. Moreover, because the revised channel-attention operator, temporal-attention branch, multiplicative fusion strategy, and cross-layer alignment were introduced together, the current comparison is package-level and cannot establish which internal CMAB modification is responsible for the observed differences. A dedicated component-level ablation that removes or adds these mechanisms individually is therefore still needed before making subcomponent-specific performance claims. For hyperparameter tuning, the matched-budget experiment showed that MFO achieved better numerical recording-level results than Random Search (RMSE 0.59 vs. 0.63 mmol/L; MARD 5.48% vs. 5.89%; Zone A 97.0% vs. 94.0%). This comparison supports the use of MFO as the tuning strategy in the present experimental setting, while not establishing that MFO is universally superior to Random Search or to other metaheuristic optimizers.

Computational feasibility also remains to be established explicitly. The conditional diffusion generator alone contains 17.46 M trainable parameters and uses a 1000-step DDPM reverse process, while the complete development pipeline additionally includes fold-specific regression training and repeated MFO candidate evaluations. Although all experiments were executed on an NVIDIA GeForce RTX 4060 Laptop GPU, wall-clock training/generation times, regression inference latency, peak memory, and energy consumption were not collected under a standardized profiling protocol. Accordingly, the present study does not establish real-time or wearable-device feasibility; future work should benchmark these quantities on representative edge hardware and investigate accelerated diffusion sampling and model compression before deployment-oriented claims are made.

An additional limitation concerns the reference-glucose acquisition metadata in the public dataset. Although the released documentation identifies the Accu-Chek Active device with calibration kit 333 and states that glucose was measured after PPG acquisition, it does not report the exact elapsed time between the two measurements, the blood specimen type, whether duplicate measurements were obtained, or within-session repeatability. These unknowns could introduce uncertainty in temporal synchronization and reference-label variability and cannot be resolved retrospectively from the released data. Accordingly, the reported prediction errors should be interpreted in the context of this reference-measurement uncertainty.

A further preprocessing limitation is the use of segment-wise z-score standardization. This choice prevents transfer of dataset-level waveform statistics between participants, but it also removes absolute baseline and amplitude information that could contain physiological signals. Because the present study did not compare segment-wise standardization with a training-derived global normalization strategy, the effect of this design choice on glucose-related information remains unresolved and should be examined in future validation.

Taken together, several limitations constrain the generalizability of the present findings. The dataset contains only 23 participants and 67 recording-level reference glucose measurements, with some participants contributing repeated recordings; participant-level clinical diagnosis was not reported, and the small cohort and restricted 4.89–10.17 mmol/L glucose range make possible demographic, physiological, and glucose-range imbalance difficult to characterize robustly. All data were obtained with a single 550 nm sensing configuration and acquisition setting, and no independent external or prospective cohort was evaluated. Although the complete five-fold evaluation was repeated with two additional participant partitions, this repeated-partition analysis is descriptive and does not remove uncertainty arising from the small sample size. In addition, CDM-generated samples remain conditioned on glucose labels already present in the outer-training data and therefore do not add new independent glucose measurements. The current dataset also does not constitute a free-living wearable validation: real-world PPG can be perturbed by motion, changes in sensor contact pressure, skin-dependent optical effects, temperature variation, and changes in peripheral perfusion. These factors, together with the absence of hypoglycemic and marked-hyperglycemic coverage, prospective validation, cross-device testing, and standardized deployment profiling, mean that the present results should be interpreted as retrospective algorithmic evidence rather than proof of clinical or wearable readiness.

## 5. Conclusions

This study presents an end-to-end framework for PPG-based non-invasive blood glucose estimation that combines physiologically conditioned diffusion augmentation, a cascaded multi-view attention BiLSTM network, and moth–flame-based hyperparameter optimization. In the primary seed-42 recording-level analysis, the proposed method achieved an RMSE of 0.59 mmol/L, an MAE of 0.38 mmol/L, a MARD of 5.48%, and Clarke Zone A and A + B proportions of 97.0% and 100%, respectively. Repeating the complete evaluation with participant-level fold assignments generated using seeds 2026 and 3407 yielded closely similar results; across the three assignments, the descriptive mean ± sample SD was 0.60 ± 0.01 mmol/L for RMSE, 0.39 ± 0.01 mmol/L for MAE, 5.52 ± 0.04% for MARD, 96.8 ± 0.2% for Clarke Zone A, and 100 ± 0.0% for Zones A + B. Secondary segment-level analyses were used only for within-study comparisons of augmentation and model variants. Overall, the results support the potential of the proposed framework for PPG-based glucose estimation in small-sample settings, while further validation on larger and more diverse cohorts remains necessary. Additional conditional analyses showed that the generated PPG waveforms preserved substantial HR-related temporal information and exhibited systematic responsiveness to changes in the BGL condition, although these analyses do not establish complete physiological equivalence. Additional quantitative validation showed that repeated generation under identical conditions produced highly consistent but non-identical PPG waveforms, while dominant frequency, peak interval, rise time, and fall time remained closely aligned with the corresponding real-signal distributions. The conditional diffusion component should be interpreted as increasing waveform variation around observed training labels rather than creating additional independent glucose measurements; the fixed 1:2 augmentation ratio and the SSIM weighting require further sensitivity and component-level validation. The reported Clarke-zone proportions should likewise be interpreted only within the observed 4.89–10.17 mmol/L glucose range and should not be taken as evidence of clinical safety or utility without external validation across broader glucose ranges and independent cohorts. Future validation should therefore include prospective external cohorts, broader glucose distributions, cross-device testing, and free-living wearable recordings that explicitly stress-test motion, contact-pressure variation, skin-dependent optical effects, temperature, and perfusion, together with standardized latency, memory, and energy measurements on target hardware.

The synthetic-to-real generalization analysis further showed that synthetic-only training was less accurate than real-only training on real held-out participants, whereas combining real and synthetic PPG yielded the best recording-level performance. This finding supports the use of conditional diffusion primarily as a training-data augmentation strategy rather than as a substitute for independent real PPG-glucose measurements.

A matched-budget hyperparameter-search comparison further showed consistently better numerical recording-level performance for MFO than for Random Search under identical search spaces and inner-validation protocols, supporting MFO as the optimization strategy used in this study without implying universal superiority over alternative optimizers.

## Figures and Tables

**Table 2:** Fold-specific hyperparameters selected independently by MFO within the five outer folds.

Outer Fold	Learning Rate (lr)	h1	h2	h3	Dropout	Attention Coefficient (λ_att)
Fold 1	0.0012	12	16	20	0.32	0.45
Fold 2	0.0009	10	14	18	0.28	0.38
Fold 3	0.0016	14	18	22	0.36	0.52
Fold 4	0.0008	8	12	16	0.25	0.31
Fold 5	0.0010	11	15	19	0.30	0.40

Note: Hyperparameters were selected independently within each outer fold using only the corresponding subject-wise inner-training (80%) and inner-validation (20%) participants. The outer-test participants and outer-test RMSE were not used for MFO fitness, hyperparameter selection, or model selection.

**Table 3:** Secondary segment-level comparison of different data augmentation strategies (mean ± SD across five outer folds).

Training Data Composition	RMSE (mmol/L)	MAE (mmol/L)	MARD (%)	Zone A (%)	Zones A + B (%)
Original data only	0.87 ± 0.04	0.67 ± 0.03	13.71 ± 0.65	86.4 ± 1.2	98.5 ± 0.8
Conventional augmentation	0.82 ± 0.04	0.64 ± 0.03	12.44 ± 0.58	88.1 ± 1.0	99.0 ± 0.5
CDM (Ours)	0.73 ± 0.03	0.58 ± 0.03	8.05 ± 0.31	90.8 ± 1.9	100.0 ± 0.0

**Table 4:** Fold-wise segment-level performance and primary pooled recording-level out-of-fold performance of the proposed full model under subject-wise five-fold cross-validation.

Outer Test Fold	RMSE (mmol/L)	MAE (mmol/L)	MARD (%)	Zone A (%)	Zones A + B (%)
Fold 1	0.69	0.51	7.72	92.59	100.0
Fold 2	0.67	0.49	7.43	96.3	100.0
Fold 3	0.70	0.53	7.86	92.31	100.0
Fold 4	0.68	0.48	7.31	96.3	100.0
Fold 5	0.65	0.51	7.59	96.3	100.0
**Fold-wise mean ± SD**	**0.68 ± 0.02**	**0.50 ± 0.02**	**7.58 ± 0.22**	**94.8 ± 2.11**	**100.0 ± 0.0**
Primary pooled recording-level OOF (67 recordings)	0.59	0.38	5.48	97.0 (65/67)	100 (67/67)

Note: Rows for Folds 1–5 and the fold-wise mean ± SD were calculated at the 5 s segment level and are retained as secondary descriptive results; they should not be interpreted as 134 independent glucose measurements. The final row is the primary seed-42 pooled recording-level analysis obtained after averaging the two 5 s out-of-fold predictions belonging to each original 10 s recording and then pooling all 67 recording-level predictions across the five held-out folds. SD, standard deviation.

**Table 5:** Secondary segment-level stepwise ablation results and package-level comparison between the original CBHFF and the improved CMAB.

Baseline	CDM	Original CBHFF	Improved CMAB	MFO	RMSE (mmol/L)	MAE (mmol/L)	MARD (%)	Zone A (%)
✓					0.87 ± 0.04	0.67 ± 0.03	13.71 ± 0.65	86.4 ± 1.2
✓	✓				0.73 ± 0.03	0.58 ± 0.03	8.05 ± 0.31	90.8 ± 1.91
✓	✓	✓			0.71 ± 0.03	0.54 ± 0.03	7.85 ± 0.31	91.6 ± 2.32
✓	✓		✓		0.71 ± 0.03	0.51 ± 0.02	7.69 ± 0.28	92.9 ± 1.86
✓	✓		✓	✓	0.68 ± 0.02	0.50 ± 0.02	7.58 ± 0.22	94.8 ± 2.11

Note: For the direct comparison, the original CBHFF and improved CMAB were evaluated using the same CDM-augmented training data and without MFO. Both models used identical convolutional channel dimensions, BiLSTM hidden-unit settings, and training configurations; the channel-attention operator, temporal-attention branch, attention-fusion strategy, and cross-layer dimension-alignment mechanism differed. All values are secondary segment-level results reported as the mean ± SD across the five subject-wise test folds and are used only for within-study model comparison. Because several CMAB changes were introduced simultaneously, this comparison quantifies only their collective package-level effect and must not be interpreted as identifying the contribution of any individual CMAB subcomponent.

**Table 7:** Descriptive cross-study context for representative blood-glucose estimation methods using PPG-containing inputs.

Model	Study Cohort/Data	Signal/Sensing Context	Glucose Range/Validation	RMSE/MARD	Reported Error-Grid Result
SFF-WICM [9]	Study-specific cohort; 21 participants; 103 days	ECG + PPG; sensor details NR	2.2–21.8 mmol/L; 10-fold CV	1.49 mmol/L/13.42%	Parkes: A 80.09%; A + B 99.49%
GBDT [8]	Multi-participant configuration; 49 volunteers	860 nm transmission PPG; perovskite photodetector	Range NR for this configuration; multi-participant evaluation	2.38 mmol/L/6.93%	Clarke: A 93%; A + B 100%
CBHFF [27]	Study-specific cohort; 21 participants; up to 6 days of PPG/BG data	PPG; sensor details NR	Glucose range NR; validation details not reproduced in the cited summary	1.67 mmol/L/17.88%	Clarke: A 61.40%; A + B 98.80%
ResNet-Transformer [31]	MIMIC-III; approximately 10,000 subjects overall	PPG (125 Hz) + historical glucose-derived inputs	3.89–13.89 mmol/L †; personalized same-subject validation	NR/11.69%	Clarke: A 82.70%; A + B ≈100%
STMF-LSTM [32]	Continuous subset: 4 non-diabetic participants; 272 samples over 16 days	625/850/940 nm differential absorbance + 940 nm PPG + previous glucose	Subset range NR; 8-fold CV by 2-day folds	0.81 mmol/L/10.12%	Parkes: A + B 100%; Zone A NR
EBTA [33]	Study-specific patient dataset; participant count NR	660/940 nm transmission PPG + HRV; pressure-controlled fingertip acquisition	Glucose range/validation NR	1.35 mmol/L/NR	Clarke: A 80.71%; A + B 100%
Ours (full model; recording-level)	Mendeley Data 37pm7jk7jn.3; 23 participants; 67 recordings	550 nm green PPG; APDS-9008 photodiode	4.89–10.17 mmol/L; participant-wise 5-fold CV; pooled recording-level OOF	0.60 ± 0.01 mmol/L/5.52 ± 0.04%	Clarke: A 96.8 ± 0.2%; A + B 100 ± 0.0%

Note: NR, not reported in the source information summarized here; CV, cross-validation; OOF, out-of-fold. Error-grid type is stated explicitly because some cited studies used Parkes rather than Clarke analysis. † The ResNet-Transformer glucose range was reported as 70–250 mg/dL and is shown here after conversion using mmol/L = mg/dL/18. The GBDT row uses the cited study’s 49-volunteer multi-participant configuration (RMSE 2.38 mmol/L, MARD 6.93%, Clarke Zone A 93%) rather than combining metrics from its separate subject-specific or sunlight experiments. The present study values are recording-level results averaged descriptively across three participant-level five-fold assignments. Because the cited studies differ in cohorts, sensing configurations, glucose ranges, validation procedures, and evaluation units, Table 7 is contextual only and is not used to infer cross-study superiority.

**Table 8:** Quantitative comparison of waveform features between real and conditionally generated PPG signals.

Feature	Real PPG	Generated PPG	Pearson’s r	MAE	Wasserstein Distance
Dominant frequency (Hz)	1.59 ± 0.17	1.59 ± 0.17	0.98 ± 0.01	0.015 ± 0.008 Hz	0.020 ± 0.010 Hz
Mean peak interval (ms)	653 ± 61	654 ± 62	0.97 ± 0.02	5 ± 3 ms	7 ± 4 ms
Rise time (ms)	201 ± 24	207 ± 25	0.91 ± 0.03	8 ± 5 ms	11 ± 6 ms
Fall time (ms)	389 ± 48	396 ± 50	0.90 ± 0.03	12 ± 7 ms	16 ± 9 ms

Note: Real and generated feature values are summarized as mean ± SD across the matched evaluation conditions. Pearson correlation, MAE, and Wasserstein distance are reported as mean ± SD across five independent generation runs.

**Table 9:** Recording-level synthetic-to-real generalization performance on real held-out participants.

Training Data	Test Data	RMSE (mmol/L)	MAE (mmol/L)	MARD (%)	Zone A (%)	Zones A + B (%)
Real only	Real held-out	0.63	0.41	5.89	95.5 (64/67)	98.5 (66/67)
Synthetic only	Real held-out	1.14	0.88	12.70	76.1 (51/67)	94.0 (63/67)
Real + Synthetic	Real held-out	0.59	0.38	5.48	97.0 (65/67)	100 (67/67)

Note: All values were calculated from the pooled 67 recording-level out-of-fold predictions under the seed-42 subject-wise five-fold partition. The outer-test data contained only real PPG recordings. Zone A and Zones A + B are also shown as counts out of 67 recording-level reference glucose measurements.

## Data Availability

The dataset analyzed in this study is publicly available in Mendeley Data, Version 3, at https://doi.org/10.17632/37pm7jk7jn.3. The primary random seed (42), the two participant-partition sensitivity seeds (2026 and 3407), and the principal-preprocessing, conditional-diffusion-model, regression-model, and MFO settings are reported in the Methods section. A reproducibility code package is provided with the revised manuscript as Supplementary File S1. The package includes participant-level outer-fold assignments, data-loading and fold-construction code, signal preprocessing, conventional augmentation, the conditional diffusion model, synthetic PPG generation, the CMAB regression model, MFO implementation and search settings, and evaluation code for RMSE, MAE, MARD, Clarke error-grid analysis, recording-level out-of-fold aggregation, and participant-clustered bootstrap analysis. No additional signal-quality rejection criterion was applied; all 67 cropped recordings were retained for subsequent segmentation and analysis. Fold-specific synthetic training waveforms are not redistributed as a separate dataset; the synthetic-generation procedure and random-seed settings are provided in the code package and Methods. The same reproducibility code package is also publicly available on GitHub at https://github.com/777588/PPG-BGL-CDM-CMAB (accessed on 22 August 2026).

## Supplementary Materials

The following supporting information can be downloaded at: https://www.mdpi.com/article/10.3390/bioengineering13090974/s1, Supplementary File S1, A reproducibility code package containing participant-level outer-fold assignments, data loading and preprocessing, conventional augmentation, conditional diffusion and synthetic-generation code, CMAB regression code, MFO optimization code, and evaluation scripts. The same reproducibility materials are publicly available at https://github.com/777588/PPG-BGL-CDM-CMAB (accessed on 22 August 2026).
